# Repurposing Synthetic Congeners of a Natural Product Aurone Unveils a Lead Antitumor Agent Inhibiting Folded P-Loop Conformation of MET Receptor Tyrosine Kinase

**DOI:** 10.3390/ph16111597

**Published:** 2023-11-13

**Authors:** Ahmed H. E. Hassan, Cai Yi Wang, Cheol Jung Lee, Hye Rim Jeon, Yeonwoo Choi, Suyeon Moon, Chae Hyeon Lee, Yeon Ju Kim, Soo Bin Cho, Kazem Mahmoud, Selwan M. El-Sayed, Sang Kook Lee, Yong Sup Lee

**Affiliations:** 1 Department of Medicinal Chemistry, Faculty of Pharmacy, Mansoura University, Mansoura 35516, Egypt; 2 Medicinal Chemistry Laboratory, Department of Pharmacy, College of Pharmacy, Kyung Hee University, 26 Kyungheedae-ro, Seoul 02447, Republic of Korea; 3 Natural Products Research Institute, College of Pharmacy, Seoul National University, Seoul 08826, Republic of Korea; 4 Department of Fundamental Pharmaceutical Sciences, Kyung Hee University, Seoul 02447, Republic of Korea; 5 Department of Pharmaceutical Chemistry, Faculty of Pharmacy, Egyptian Russian University, Badr City 11829, Egypt

**Keywords:** natural products congeners, aurone, sulfuretin, antitumor agents, colon cancer, cell cycle arrest, apoptosis, MET kinase

## Abstract

A library of 24 congeners of the natural product sulfuretin were evaluated against nine panels representing nine cancer diseases. While sulfuretin elicited very weak activities at 10 µM concentration, congener **1t** was identified as a potential compound triggering growth inhibition of diverse cell lines. Mechanistic studies in HCT116 colon cancer cells revealed that congener **1t** dose-dependently increased levels of cleaved-caspases 8 and 9 and cleaved-PARP, while it concentration-dependently decreased levels of CDK4, CDK6, Cdc25A, and Cyclin D and E resulting in induction of cell cycle arrest and apoptosis in colon cancer HCT116 cells. Mechanistic study also presented MET receptor tyrosine kinase as the molecular target mediating the anticancer activity of compound **1t** in HCT116 cells. In silico study predicted folded p-loop conformation as the form of MET receptor tyrosine kinase responsible for binding of compound **1t**. Together, the current study presents compound **1t** as an interesting anticancer lead for further development.

## 1. Introduction

Globally, cancer is the second leading cause of death amongst people in developed and developing countries [1,2,3,4,5]. In fact, cancer refers to a group of diseases characterized by common hallmarks including hyperproliferation, stress response survival, angiogenesis, invasive growth, metastasis, and metabolic reprogramming, as well as an altered microenvironment and immune response [6,7,8]. Being a complex multifactorial disease, cancer treatments employing multifunctional molecules targeting more than one disease component might be more effective and prone to evolution of resistance [9,10,11,12,13,14].

Inflammation is an axial component in cancer diseases. While acute inflammation response can promote tumor suppression, chronic inflammation was found to create an immunosuppressive microenvironment [15,16,17,18,19,20,21,22]. In addition, several inflammatory mediators and signaling pathways such as PGE_2_, cytokines, and NF-κB promote survival, invasion, and metastasis of cancer cells [15]. Consequently, targeting inflammation signaling pathways and production of inflammatory mediators has been clinically included in cancer therapy [15]. 

Proliferation of cells follows sequential steps of cell cycle phases that involve duplication of cells’ DNA and organelles followed by cell division. Several signaling pathways are involved in initiation and regulation of these phases. Within cancer cells, genetic mutations and/or epigenetic factors usually results in dysregulation of pathways promoting and/or repressing cell cycle and proliferation [23,24,25,26,27,28,29,30,31,32]. Therefore, agents interrupting and interfering with the cell cycle are helpful antitumor agents stalling the growth of cancers tissues. In addition, programmed cell death, which is a cell’s active self-destruction that might be triggered by apoptosis, necrosis, or autophagy, is a mechanism that several anticancer agents can trigger, inducing death in cancer cells [33,34,35,36]. Such programmed cell death occurs in response to several signaling pathways and, thus, agents that impact these pathways could be useful anticancer agents. 

Since ancient history, nature has served as a major source for drug discovery and development. Indeed, natural-product-based drug discovery affords higher success rates [37,38,39,40,41]. The fact that natural products are privileged structures might be the reason for these higher success rates. In lieu of the above-mentioned literature reports, we have embarked on natural-product-based drug discovery towards the development of multifunctional molecules with anti-inflammatory and antiproliferative activity. Herein, we report our approach and results.

## 2. Results and Discussion

### 2.1. Rational of Profiling a Focused Library of Sulfuretin-Congeners as Anticancer Agents

**Figure 1:**
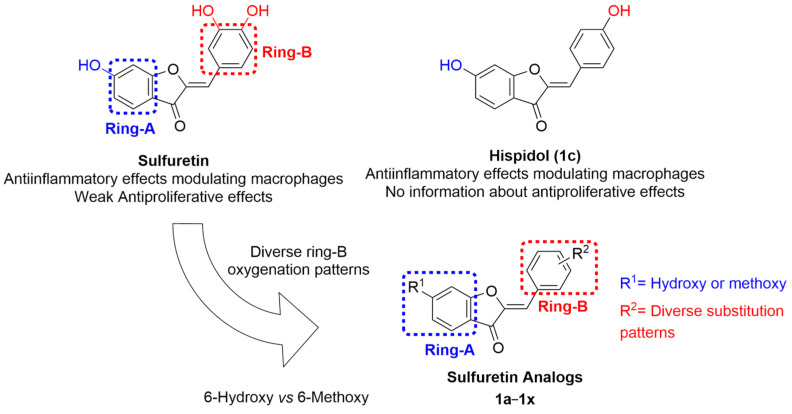
Design of sulfuretin’s congeners focused library for profiling anticancer activities.

Sulfuretin (Figure 1), an aurone bearing *meta* and *para*-dihydroxy substituents at ring-B and a *C*^6^-hydroxy moiety at ring-A, is a natural product obtained from *Rhus verniciflua* which exhibits interesting properties [42]. Interestingly, sulfuretin was found to induce apoptosis through activation of Fas, Caspase-8, and the mitochondrial death pathway [42]. In addition, sulfuretin was reported to elicit anticancer effects, inhibiting cell invasion through inhibition of NF-κB which triggers downregulation of MMP-9 expression [43]. Despite the interesting anticancer activity of sulfuretin, these reports are limited, as they incorporated a minimal number of cell lines and, thus, would be far from providing sufficient data on the anticancer spectrum and potency of sulfuretin.

In addition to the antiproliferative activity, sulfuretin was also reported to show potential anti-inflammatory effects through inhibition of the NF-κB signaling pathway, a common pathway for inflammation and cancer [44]. However, hispidol (Figure 1), a congener of sulfuretin, was found to be a more potent inhibitor of macrophage production of PGE_2_, an inflammatory mediator that is correlated with both inflammation and cancer [45,46]. Nevertheless, there is no reported study, to the best of our knowledge, on possible anticancer effects of the sulfuretin congener, hispidol.

Considering the above-mentioned information, the current work was planned to address the profiling of the anticancer activity of sulfuretin against nine panels including diverse cancer cell lines belonging to nine cancer diseases of different origins to explore its anticancer spectrum and potency. Considering that different oxygenation patterns in the form of hydroxy and/or methoxy moieties is a common structural feature in natural products, a library of twenty-four sulfuretin congeners bearing diverse oxygenation patterns on ring-B and a *C*^6^-hydroxy or methoxy moiety on ring-A (Figure 1), and reported anti-inflammatory activity, were selected for profiling their anticancer spectrum in an attempt to obtain insights into SAR against the same panels of diverse cancers used for sulfuretin. The list of the compounds as well as their reported anti-inflammatory effects are presented in Table 1.

### 2.2. Profiling of Sulfuretin and Its Congeners for Antiproliferative Activities

#### 2.2.1. Blood Cancers

**Figure 2:**
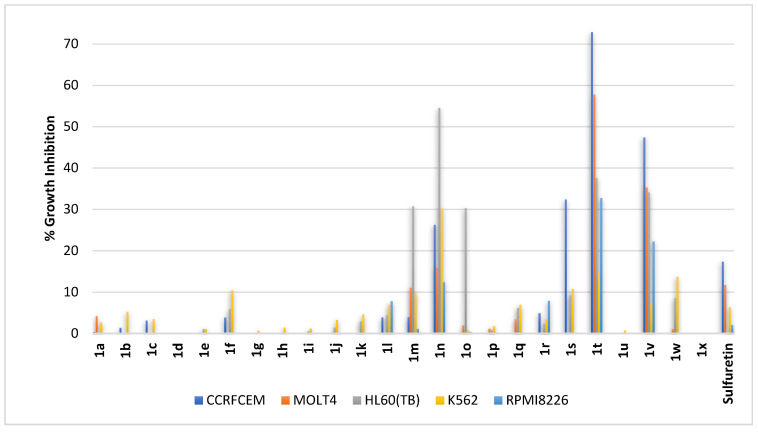
% growth inhibition of diverse blood cancer cell lines triggered by 10 µM concentrations of sulfuretin and its congeners (**1a–1x**).

Blood cancers are a group of diverse cancers that may originate from lymphoblastic or myeloid progenitor cells and, furthermore, might be acute or chronic. Towards near inclusive evaluation, five cell lines were employed including: (1) CCRFCEM, a childhood T acute lymphoblastic leukemia (ALL); (2) MOLT4, an acute lymphoblastic leukemia (ALL); (3) HL60(TB), an acute myeloid leukemia (AML); (4) K562, a chronic myelogenous leukemia (CML); and (5) RPMI8226, a multiple myeloma (MM). At the tested concentration, sulfuretin showed very limited activity against CCRFCEM and MOLT4 (Figure 2); both are acute lymphoblastic leukemia, ALL. It was almost ineffective against the employed cell lines of myeloid origin (HL60(TB), K562, and RPMI8226). In contrast, the most effective compound was derivative **1t** combining polar, hydrogen-bond-donor or acceptor dihydroxy moieties at vicinal *ortho*- and *meta*-positions of ring-B with the more steric, less polar hydrogen-bond-donor-only methoxy moiety at the *C*^6^-position of ring-A (Figure 2). It was most effective against the ALL cell lines CCRFCEM and MOLT4, triggering growth inhibition near 73% and 58%, respectively. It was less active against cell lines of myeloid origin, especially the CML and K562 cell lines. Shifting the dihydroxy moieties to vicinal *meta*- and *para*-positions of ring-B of compound **1t** to afford derivative **1v** resulted in lowered activity (Figure 2), but loss of activity was found for derivative **1u** upon placing the dihydroxy moieties at *ortho*- and *para*-positions of ring-B, i.e., the ring carbons bearing the dihydroxy moieties were separated by one carbon. In comparison, all compounds **1d–1e** having dihydroxy moieties at ring-B but possessing a *C*^6^-hydroxy moiety on ring-A were of low to no activity at the tested concentration regardless of the position of the dihydroxy moieties on ring-B. Compounds incorporating only one hydroxy moiety on ring-B combined with the *C*^6^-methoxy moiety on ring-A (compounds **1q–1s**) or *C*^6^-hydroxy moiety on ring-A (compounds **1a–1c**), derivatives having a methoxy moiety on ring-B combined with *C*^6^-hydroxy moiety on ring-A (compounds **1h–1j**) or *C*^6^-methoxy moiety on ring-A (compounds **1w** and **1x**), and trihydroxylated or trimethoxylated ring-B derivatives (compounds **1g** and **1o**) were found to be almost inactive at the tested concentration. Such an outcome, coupled with the found low to no activity of compounds **1k–1m** possessing dimethoxy moieties at ring-B combined with a *C*^6^-hydroxy moiety on ring-A, might support the conclusion that the vicinal dihydroxy moieties at *ortho*- and *meta*-positions of ring-B combined with a *C*^6^-methoxy moiety on ring-A might be optimal for activity. However, compound **1n** possessing dimethoxy moieties at both *meta*-positions of ring-B showed potential activity. Interestingly, its activity was higher on HL60(TB) of myeloid origin; this represents a distinct pattern that is different from compounds **1t** and **1v** which were more active on cell lines of lymphoblastic origin. 

#### 2.2.2. Non-Small-Cell Lung Cancer

**Figure 3:**
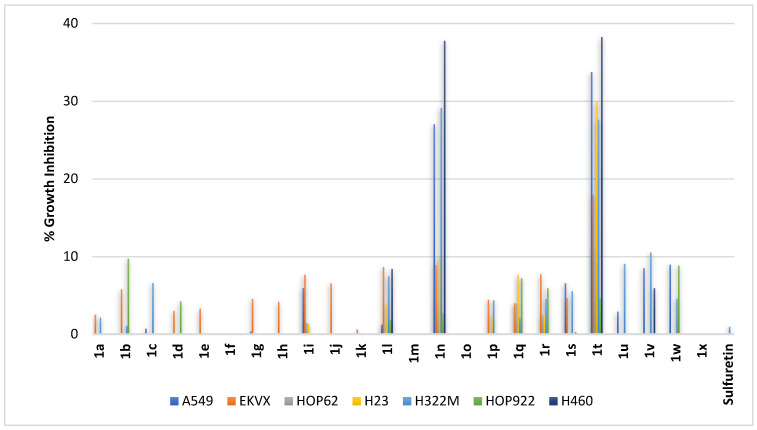
% growth inhibition of diverse non-small-cell lung cancer cell lines triggered by 10 µM concentrations of sulfuretin and its congeners (**1a–1x**).

Non-small lung cancer involves multiple subtypes. Accordingly, compounds **1a–1x** were profiled against lung adenocarcinoma cells (A549, EKVX, HOP62, and H23), bronchoalveolar carcinoma cells (H322M), and large-cell lung carcinoma cell lines (HOP92 and H460). As shown in Figure 3, an almost null activity was elicited by sulfuretin at the tested concentration. In contrast, two potential compounds were identified (Figure 3), which were compound **1t** combining polar, hydrogen-bond-donor or acceptor dihydroxy moieties at vicinal *ortho*- and *meta*-positions of ring-B with the more steric, less polar hydrogen-bond-donor methoxy moiety at *C*^6^-position of ring-A and compound **1n** having a *C*^6^-hydroxy moiety ring-A but possessing the less polar dimethoxy moieties at both *meta*-positions of ring-B. Both compounds elicited almost the same level of activity against the bronchoalveolar carcinoma cell line H322M and the human large-cell lung carcinoma cell line H460, but not HOP92. However, compound **1t** was more active against lung adenocarcinoma cell lines A549, EKVX, HOP62, and H23, suggesting different molecular targets impacted by compounds **1t** and **1n**. Except for compounds **1t** and **1n**, the activity was limited for all other compounds whether bearing a *C*^6^-hydroxy moiety at ring-A combined with hydroxy or methoxy moieties on ring-B (compounds **1a–1g** and **1h–1p**, respectively; Figure 3) or bearing a *C*^6^-methoxy moiety at ring-A combined with hydroxy or methoxy moieties on ring-B (compounds **1q–1v**, **1w**, and **1x**; Figure 3). 

#### 2.2.3. Colorectal Cancer

**Figure 4:**
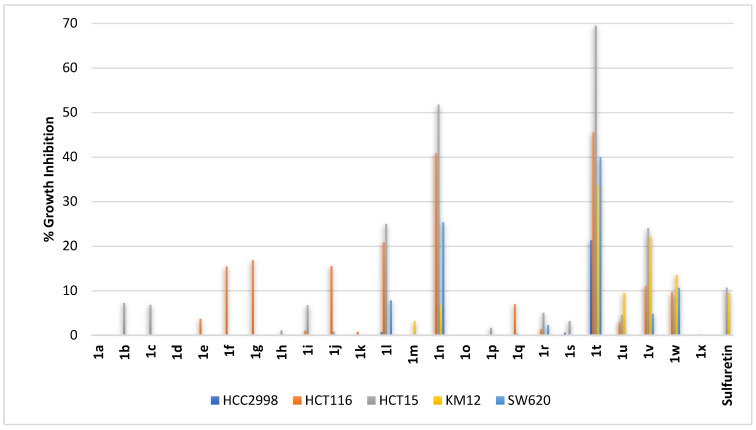
% growth inhibition of growth of diverse colorectal cancer cell lines triggered by 10 µM concentrations of sulfuretin and its congeners (**1a–1x**).

Colon adenocarcinoma is the most common type of colon cancers. Consequently, sulfuretin and its congeners were profiled against five colorectal adenocarcinoma cell lines (HCC2998, HCT116, HCT15, KM12, and SW620). In regards to sulfuretin, the growth of only two cell lines (HCT15 and KM12) was weakly inhibited by almost 10% while no inhibition was observed at all against the other three cell lines at the tested concentration (Figure 4). In consensus with the activity pattern revealed from profiling sulfuretin congeners against non-small lung cancers, the results showed that compounds **1t** and **1n** were the most active. Out of the five cell lines used, the highest growth inhibition activities were measured against HCT116 and HCT15 cell lines (Figure 4). It became clear from profiling results that, in general, all other compounds, whether bearing a *C*^6^-hydroxy moiety at ring-A combined with hydroxy or methoxy moieties on ring-B (compounds **1a–1g** and **1h–1p**, respectively; Figure 4) or bearing a *C*^6^-methoxy moiety at ring-A combined with hydroxy or methoxy moieties on ring-B (compounds **1q–1v**, **1w** and **1x**; Figure 4), are very weak growth inhibitors for the tested colorectal cancer cell lines. It might be concluded that the structural features of compounds **1t** and **1n** render them possible hit compounds. 

#### 2.2.4. Brain Cancer 

**Figure 5:**
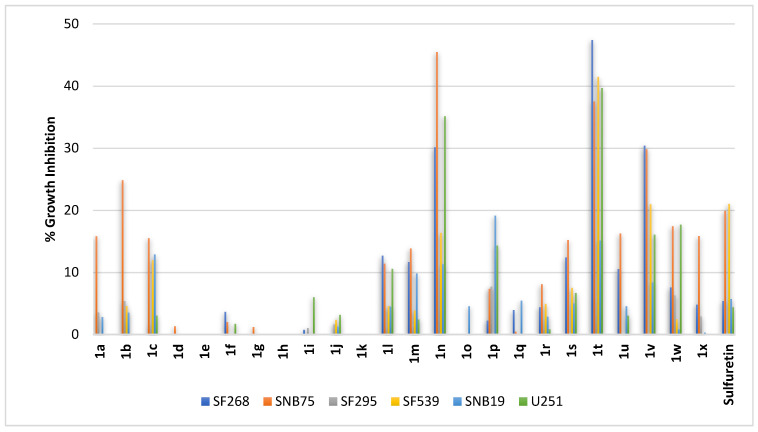
% growth inhibition of growth of diverse CNS cancer cell lines triggered by 10 µM concentrations of sulfuretin and its congeners (**1a–1x**).

Gliomas are common brain cancers that originate from glial cells. Considering the hematopoietic origin of glial cells [47,48], it might be understandable that profiling results of sulfuretin and its congeners against GBs showed some similarities to the activity pattern against blood cancers rather than against lung and colon cancers. Because of the heterogeneity of gliomas, compounds **1a–1x** were profiled against diverse cells including SF268, SNB75, SF295, SF539, SNB19, and U251. As shown in Figure 5, sulfuretin triggered around 20% growth inhibition of astrocytoma cell line SNB75 and glioblastoma cell line SF539 but showed no inhibition at all for glioblastoma multiforme cell line SF295 and only 5% inhibition of anaplastic astrocytoma cell line SF268 and glioblastoma cell lines SNB19 and U251. Meanwhile, the most active amongst the tested compounds was compound **1t**, bearing dihydroxy moieties at vicinal *ortho*- and *meta*-positions of ring-B coupled with the less polar hydrogen-bond-donor-only methoxy moiety at the *C*^6^-position of ring-A. It showed almost 10-fold the activity of sulfuretin against the anaplastic astrocytoma SF268 cell line, triggering near 50% growth inhibition. In addition, it induced near 40% growth inhibition of SF539 and U251 glioblastoma cell lines and the SNB75 astrocytoma cell line. Like the activity pattern against blood cancers, compound **1v**, incorporating a *C*^6^-methoxy moiety at ring-A as in compound **1t** but with the dihydroxy moieties shifted to vicinal *meta*- and *para*-positions of ring-B, showed significant, yet less, activity relative to compound **1t** against these four cell lines (Figure 5). Other compounds sharing the *C*^6^-methoxy moiety at ring-A of compounds **1t** and **1v** were of much lower activities, regardless of incorporating other hydroxylation or methoxylation patterns on ring-B (compounds **1q–1s**, **1u**, **1w**, and **1x**; Figure 5). Out of the compounds having a *C*^6^-hydroxy moiety on ring-A, compound **1n** possessing dimethoxy moieties at both *meta*-positions of ring-B showed potential activity (Figure 5). It was most active, in order, against the SNB75 astrocytoma cell line, U251 glioblastoma cell line, and SF268 anaplastic astrocytoma cell line. Meanwhile, compounds bearing dimethoxy moieties at the distal *ortho*- and *meta*-positions or *meta*- and *para*-positions of ring-B (compounds **1l** and **1m**, respectively) or bearing a monohydroxy moiety at ring-B (compounds **1a–1c**) showed low activity. All other tested derivatives of the *C*^6^-hydroxy series bearing dihydroxy, polyhydroxy or monomethoxy substituents on ring-B were virtually inactive. Collectively, the results suggest compounds **1t**, **1v**, and **1n** as possible hits possessing significant activities against gliomas. 

#### 2.2.5. Skin Cancer

**Figure 6:**
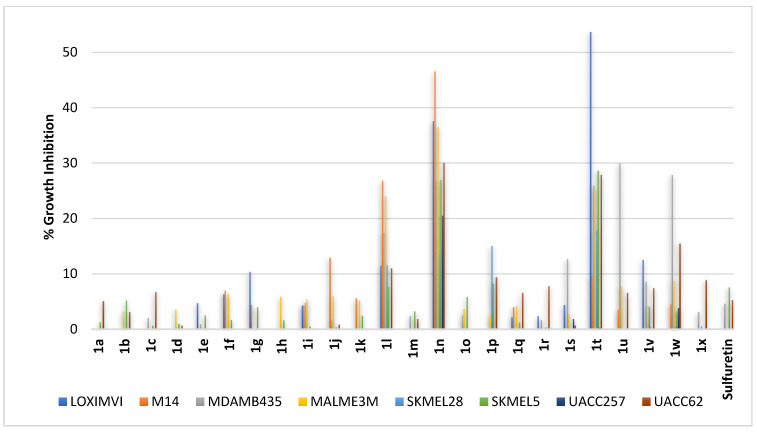
% growth inhibition of growth of diverse melanoma cancer cell lines triggered by 10 µM concentrations of sulfuretin and its congeners (**1a–1x**).

Melanoma is the most aggressive form of skin cancer. Accordingly, compounds **1a–1x** were profiled against diverse melanoma cells, including the following: LOXIMVI, M14, MDAMB435, MALME3M, SKMEL28, SKMEL5, UACC257, and UACC62. As revealed from profiling results, sulfuretin was almost ineffective. It showed no inhibition for five cell lines out of the employed eight cell lines and, furthermore, the growth inhibition found for the other three cell lines was less than 8%. In line with the activity pattern found against lung cancers, compounds **1t** and **1n** were the most active derivatives (Figure 6). The metastatic amelanotic melanoma LOXIMVI was the cell line most affected by compound **1t**, showing a growth inhibition of more than 50%. Compound **1t** also triggered significant growth inhibition for all other tested melanoma cell lines except for the UACC257 cell line (Figure 6). However, the UACC257 cell line was significantly inhibited by compound **1n** and the metastatic amelanotic melanoma M14 cell line was the most impacted by this compound (Figure 6). Considering the different activity profiles of compounds **1t** and **1n** coupled with structural feature differences, it might be inferred that different molecular targets might be involved in mediating the antiproliferative activities of each of them. Other than compound **1t**, derivatives sharing the *C*^6^-methoxy moiety on ring-A and bearing hydroxy or methoxy moieties on ring-B (compounds **1q–1v**, **1w**, and **1x**; Figure 6) showed limited activities in general. Other than compound **1n**, almost all compounds having the *C*^6^-hydroxy moiety on ring-A and bearing hydroxy or methoxy moieties on ring-B (compounds **1a–1p**) showed almost no significant activity except for compound **1l** that showed low activity against most of the employed melanoma cell lines. 

#### 2.2.6. Ovarian Cancer

**Figure 7:**
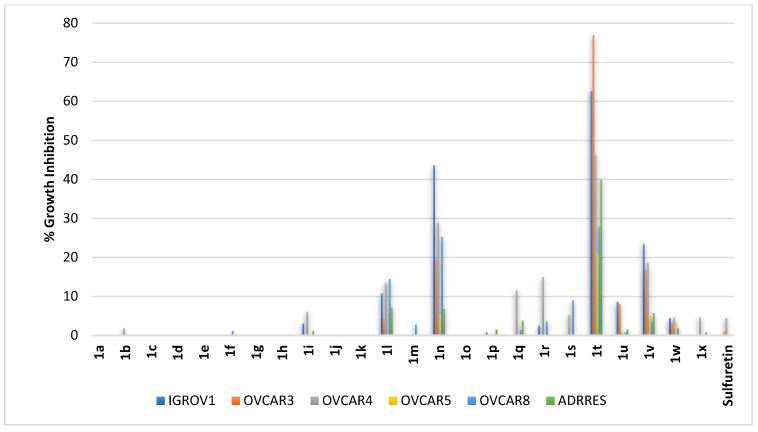
% growth inhibition of growth of ovarian cancer cell lines triggered by 10 µM concentrations of sulfuretin and its congeners (**1a–1x**).

High-grade serous ovarian carcinoma (HGSOC) is the most common ovarian cancer. Meanwhile, endometrioid carcinoma is an invasive ovarian cancer. Accordingly, compounds **1a–1x** were profiled against IGROV1 (an endometrioid carcinoma), as well as OVCAR3, OVCAR4, OVCAR5, OVCAR8, and ADRRES (all are HGSOC cells). The results showed that sulfuretin was almost ineffective against all employed cell lines. As shown in Figure 7, potential activity was clear for compound **1t** which triggered almost 77% growth inhibition of the high-grade ovarian serous adenocarcinoma OVCAR3 cell lines and near 63% growth inhibition of the endometrioid carcinoma IGROV1 cell line. It also inhibited other high-grade ovarian serous adenocarcinoma cell lines by varying degrees. Other compounds sharing the *C*^6^-methoxy moiety on ring-A present in compound **1t** but with other hydroxy or methoxy substitution patterns on ring-B (compounds **1q–1x**) were almost inactive except for minor activity for compound **1v**. Except for some activity for compound **1n** bearing a dimethoxy substitution pattern at both *meta*-positions of ring-B and, to a lesser extent, compound **1l** possessing a dimethoxy substitution pattern at distal *ortho*- and *meta*-positions of ring-B, all other compounds with the *C*^6^-hydroxy moiety on ring-A were almost inactive (compounds **1a–1p**, Figure 7). 

#### 2.2.7. Renal Cancer

**Figure 8:**
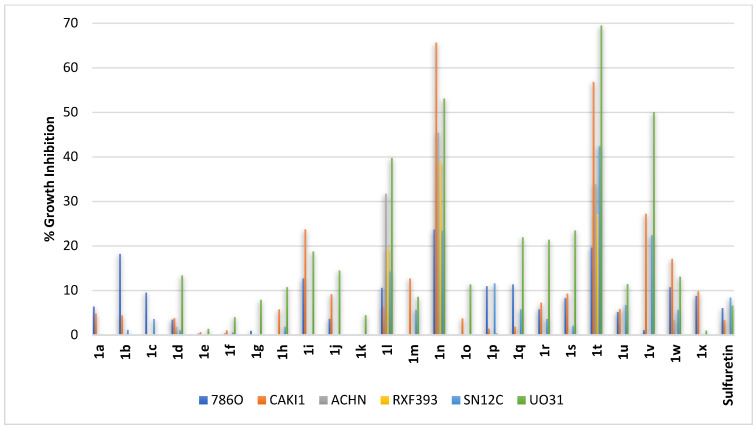
% growth inhibition of growth of diverse renal cancer cell lines triggered by 10 µM concentrations of sulfuretin and its congeners (**1a–1x**).

Renal cell carcinomas (RCC) could be clear-cell renal cell carcinomas (ccRCC; the most common type) or non-clear-cell renal cell carcinomas (nccRCC; heterogeneous and difficult) [49]. Consequently, compounds **1a–1x** were profiled against a panel composed of the following: (1) 786O, a primary ccRCC cell line; (2) CAKI1, a metastatic ccRCC cell line; (3) ACHN, a metastatic papillary renal cell carcinoma (a subtype of nccRCC); (4) RXF393, an unclassified poorly differentiated renal cell carcinoma; (5) SN12C, an unclassified renal cell carcinoma; and (6) UO31, an unclassified renal cell carcinoma. As illustrated in Figure 8, sulfuretin elicited very weak to no growth inhibition against all types of renal cancer cell lines used. In general, weak to no growth inhibition was the outcome for all derivatives with a *C*^6^-hydroxy moiety on ring-A coupled with hydroxy functions on ring-B (compounds **1a–1g**; Figure 8) or with methoxy functions on ring-B (compounds **1h–1p**; Figure 8), except for compound **1n** and to a lesser extent compound **1l**. Such an activity profile resembles the profiles against colon, melanoma, and ovarian cancers that also showed an increased activity from compound **1n** and to a lesser extent compound **1l**. The only structural difference between compounds **1n** and **1l** is that one of the dimethoxy moieties occupying both *meta*-positions of ring-B in compound **1n** is shifted to the *ortho*-position distant from the other *meta*-methoxy in compound **1l**. The cell lines most inhibited by compound **1n** were the metastatic ccRCC CAKI1 cell line, the unclassified renal cell carcinoma UO31 cell line, and, to a lesser extent, the metastatic papillary renal cell carcinoma ACHN cell line. Amongst the compound series sharing the *C*^6^-methoxy moiety on ring-A, compound **1t** elicited potential activity which is in line with the activity profile found against colon, melanoma, and ovarian cancers. Similar to compound **1n**, the most affected cell lines for compound **1t** were the metastatic ccRCC CAKI1 cell line and the unclassified renal cell carcinoma UO31 cell line. Other compounds with a *C*^6^-methoxy moiety on ring-A combined with hydroxy functions on ring-B (compounds **1q–1v**; Figure 8) or with methoxy functions on ring-B (compounds **1w** and **1x**; Figure 8) were, in general, much less active, except for a significant inhibition of the unclassified renal cell carcinoma UO31 cell line by compound **1v**. Collectively, it might be concluded that the structural features of compounds **1t** and **1n** suggest them as hit compounds for further development of more potential renal cancer therapeutics. 

#### 2.2.8. Prostate Cancer

**Figure 9:**
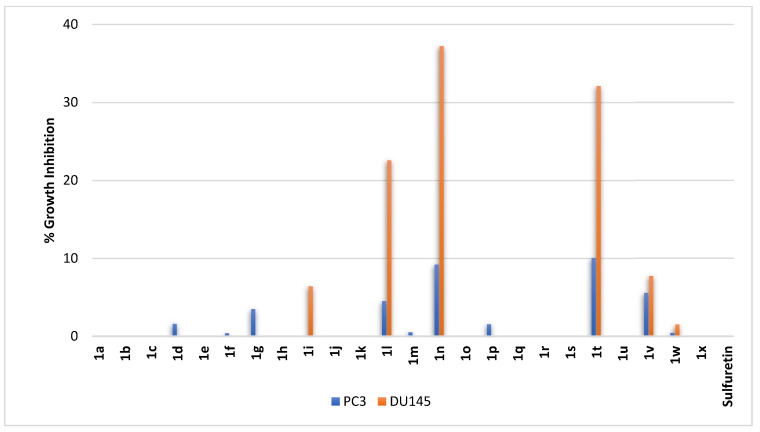
% growth inhibition of growth of prostate cancer cell lines triggered by 10 µM concentrations of sulfuretin and its congeners (**1a–1x**).

Compounds **1a–1x** were profiled against two metastatic prostate adenocarcinoma cell lines, PC3 and DU145. As shown in Figure 9, sulfuretin was completely ineffective at the tested concentration against both cell lines. Similarly, all compounds **1a–1g** having hydroxy groups on ring-B in conjunction with a *C*^6^-hydroxy moiety on ring-A had negligible to no effect on both metastatic DU145 and PC3 cell lines. Replacement of the hydroxy substitution patterns on ring-B by methoxy substitution patterns enabled three compounds to trigger growth inhibition of the metastatic DU145 cell line (**1i**, **1l**, and **1n**, Figure 9). These three compounds have a common methoxy group in the *meta*-position on ring-B. The most effective amongst them, compound **1n**, has another methoxy moiety at the second *meta*-position of ring-B; the least active, compound **1i**, has no other methoxy moiety on ring-B, while the intermediately active compound **1l** possesses a second methoxy group shifted by one carbon relative to that of compound **1n** to occupy the *ortho*-position distant from first methoxy *meta*-position on ring-B. However, compounds **1k** and **1m** with a methoxy moiety at *meta*-position of ring-B and a second methoxy moiety at the vicinal *ortho*- or *para*-positions on ring-B were inactive. It might be inferred from this that a molecular target in the DU145 cell line, but absent or less expressed in the PC3 cell line, is impacted by compounds with a methoxy moiety at the *meta*-position on ring-B coupled with a *C*^6^-hydroxy function on ring-A. It might be also concluded that interactions with such molecular targets are increased by the presence of a second methoxy moiety at the other *meta*-position and to a lesser degree at the distant *ortho*-position, but not the *para*- or the vicinal *ortho*-position. On the other hand, compounds **1t** and **1v** possessing dihydroxy moieties on ring-B where one of these hydroxy groups were at the meta-position of ring-B were the active compounds amongst derivatives having a *C*^6^-methoxy moiety on ring-A. Out of them, compound **1t**, having the dihydroxy groups at the vicinal *ortho*- and *meta*-positions of ring-B, was more active than compound **1v**, itself having the dihydroxy groups at the vicinal *meta*- and *para*-positions (Figure 9). Compounds **1n** and **1t** might possibly be nominated as hit compounds inhibiting the metastatic DU145 prostate adenocarcinoma cell line. 

#### 2.2.9. Breast Cancer

**Figure 10:**
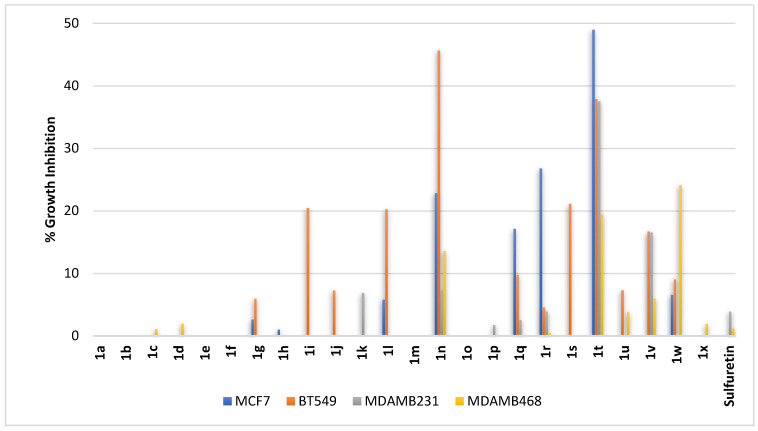
% Growth inhibition of growth of diverse breast cancer cell lines triggered by 10 µM concentrations of sulfuretin and its congeners (**1a–1x**).

Breast cancer could be hormone-sensitive or resistant and, in addition, primary or metastatic. Accordingly, compounds **1a–1x** were profiled against four breast cancer cells including the following: (1) MCF7, a metastatic invasive hormone-responsive breast adenocarcinoma; (2) BT549, a primary triple-negative invasive breast adenocarcinoma; (3) MDAMB231, a metastatic triple-negative breast adenocarcinoma; and (4) MDAMB468, a metastatic triple-negative breast adenocarcinoma. As shown in Figure 10, sulfuretin had almost negligible to no activity against the cell lines employed. As revealed from the results, the profile of sulfuretin congeners’ activity against the primary triple-negative BT549 cell line was distinct from the activity profile against the metastatic hormone-responsive MCF7 cell line and metastatic MDAMB231 and MDAMB468 triple-negative cell lines. In general, all *C*^6^-hydroxy derived compounds having hydroxy substituents on ring-B did not inhibit the growth of any primary or metastatic cell lines (compounds **1a–1g**, Figure 10). Meanwhile, out of the *C*^6^-hydroxy derived compounds **1h–1p** having methoxy substituents at ring-B, compounds **1i**, **1l**, and **1n** with at least one methoxy substituent at *meta*-position of ring-B or compound **1j** with one methoxy substituent at the *para*-position of ring-B triggered growth inhibition of the primary triple-negative BT549 cell line (Figure 10). Amongst them, the most effective was compound **1n**, possessing two methoxy moieties at both *meta*-positions of ring-B, while the equally active compounds **1i** and **1l** have either only one methoxy moiety at *meta*-position of ring-B or the second methoxy moiety shifted by one carbon relative to that of compound **1n** to occupy the *ortho*-position distant from first methoxy *meta*-position on ring-B. This indicates that a methoxy moiety at *ortho*-position of ring-B has no role in mediating the activity, which might be supported by the found no activity of compound **1h** with one methoxy moiety at *ortho*-position of ring-B. In the case of the least active compound **1j**, it has only one methoxy moiety but shifted to the *para*-position of ring-B. Accordingly, it might be inferred that the methoxy moiety at *meta*-position is more optimal for activity against the primary triple-negative BT549 cell line. In the case of metastatic cell lines, all *C*^6^-hydroxy derived compounds **1h–1p** with methoxy substituents at ring-B did not trigger significant growth inhibition except for compound **1n**, possessing two methoxy moieties at both *meta*-positions of ring-B, that showed some inhibition of the metastatic hormone-responsive MCF7 cell line. However, some members of *C*^6^-methoxy derived compounds **1q–1v**, having hydroxy substituents at ring-B, showed potential inhibition of the metastatic hormone-responsive MCF7 cell line (Figure 10). As revealed from the results, this antiproliferative activity against the metastatic hormone-responsive MCF7 cells might be associated with hydroxy moieties at the *ortho*- and *meta*-positions of ring-B (compounds **1q** and **1r**, respectively). When both vicinal *ortho*- and *meta*-positions were substituted simultaneously by dihydroxy moieties, the activity was more enhanced (compound **1t**). In addition, compound **1t**, amongst the *C*^6^-methoxy derived compounds **1q–1x**, showed the best inhibition of the growth of the primary triple-negative BT549 cell line and metastatic triple-negative MDAMB231 cell line. However, the growth of the triple-negative MDAMB468 cell was more inhibited by compound **1w**, possessing a *ortho*-methoxy substituent on ring-B relative to compound **1t** possessing vicinal *ortho*- and *meta*-methoxy substituents on ring-B. Such a difference in structural feature requirements suggests the involvement of different molecular targets. Collectively, the results might nominate compound **1t** as a hit candidate for the development of antiproliferative compounds against metastatic hormone responsive MCF7 or triple-negative primary and metastatic triple-negative BT549 and MDAMB231 breast cancers. In addition, compound **1n** might be nominated as a hit compound against primary triple-negative BT549 cells. 

### 2.3. Compound 1t Induces Cell Cycle Arrest in HCT116 Colon Cancer Cells

To assess the mechanism mediating the potential antiproliferative activity of compound **1t**, the potency of compound **1t** was first assessed against three cell lines that were available to us and included in the panels used to profile the activity that involved lung cancer A549, colon cancer HCT116, and breast cancer MDAMB231 cell lines. As shown in Table 2, compound **1t** showed potent inhibitory activity for HCT116 cell growth with a low micromolar IC_50_ value in the range of 8.68 μM. Consequently, the mechanism of action of compound **1t** was studied employing colon cancer HCT116 cells.

**Figure 11:**
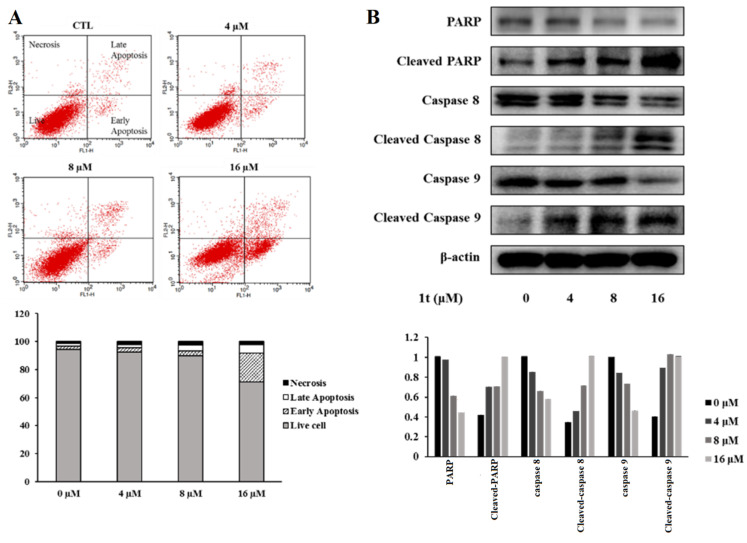
Apoptotic effects of compound **1t** in HCT116 cells. (**A**) Flow cytometry after staining with annexin V-FITC/PI; (**B**) PARP, cleaved PARP, caspase 8, cleaved caspase 8, caspase 9, and cleaved caspase 9 protein expressions in compound **1t**-treated HCT116 cells.

Because sulfuretin was reported to induce apoptosis through activation of Fas, Caspase-8, and the mitochondrial death pathway in HL60(TB) blood cancer [42], a flow cytometry assay was addressed for compound **1t** in HCT116 cells at different doses to explore possible apoptotic and/or necrotic activity. The results showed that compound **1t** could significantly induce the apoptosis in HCT116 cells (Figure 11A). Accordingly, Western blotting was addressed to assess proteins involved in apoptosis. The results showed that compound **1t** dose-dependently increased the levels of cleaved-caspase 8 and 9 and cleaved-PARP and significantly decreased levels of caspase 8, and 9 as well as PARP in a dose-dependent manner in HCT116 cells (Figure 11B). These results further confirmed that compound **1t** can induce apoptotic death of HCT116 cells. Furthermore, assessment of cycle distribution was addressed to explore possible correlation of the antiproliferative activities of compound **1t** with cell cycle arrest. 

**Figure 12:**
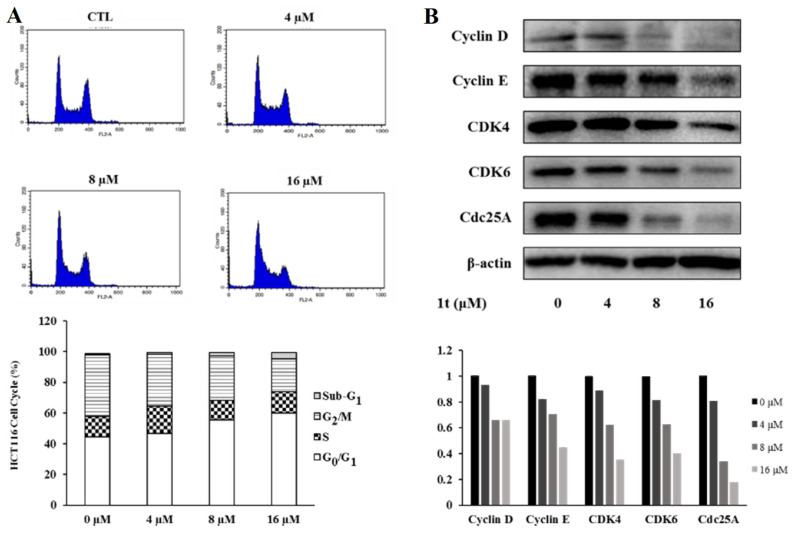
Impact of compound **1t** on the cell cycle distribution in HCT116 cells. (**A**) HCT116 cell cycle distributions after treatment with different concentrations of compound **1t**. The graphs show the quantified results. (**B**) CDK4, CDK6, Cyclin D, Cyclin E, and Cdc25A protein expressions after treatment with different concentrations of compound **1t**.

Using variable concentrations of compound **1t**, analysis of HCT116 cell distribution in the different phases of cell cycle progression revealed that compound **1t** dose-dependently increased the G_0_/G_1_ cell population (Figure 12A). Therefore, Western blotting was addressed to assess changes in levels of proteins involved in cell cycle regulation. In line with cell cycle analysis results, compound **1t** was found to suppress CDK4, CDK6, Cdc25A, and Cyclin D and E protein levels in a concentration-dependent manner (Figure 12B). Together, these results indicate that the antiproliferative effects of the compound **1t** are associated with G_0_/G_1_ cell cycle arrest. 

### 2.4. Compound 1t Inhibits Activation of MET Receptor Tyrosine Kinase and Downregulates Its Downstream in HCT116 Colon Cancer Cells

Activation of phosphatidylinositol 3-kinase (PI3K)/Akt/mammalian target of rapamycin (mTOR) pathways is involved in cancer cells’ increased motility, proliferation, migration, invasion, survival, and angiogenesis [50,51,52]. It was reported that the PI3K/AKT/mTOR intracellular pathway plays a key role in the regulation of the cell cycle in colon cancer [53]. Their biochemical function is diametrically related to cellular quiescence, metastasis, tumor, and lifespan [54,55]. PI3K/Akt/mTOR pathways have been elucidated for their biofunctional involvement in colorectal tumorigenesis. As clinical pathological biological features, the abnormal regulation of these signals has frequently been detected with concomitant overexpression of related proteins. Interestingly, these cellular signaling pathways are downstream components of the MET signaling pathway. These observations and the elucidated role of MET signaling pathways in the invasive growth of cancer have enhanced enthusiasm for the development of MET inhibitors as anticancer cancer agents. 

**Figure 13:**
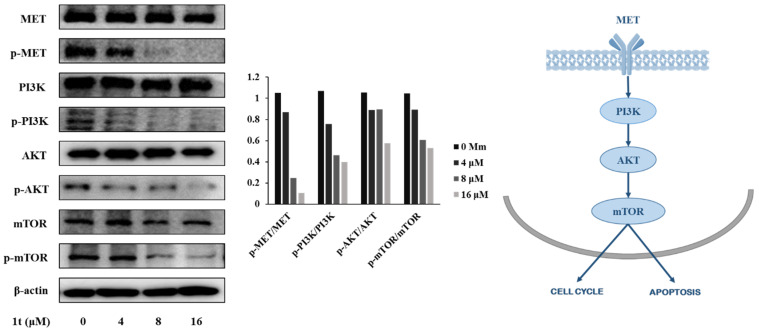
Impact of compound **1t** on MET/PI3K/AKT/mTOR pathway in HCT116 cells.

Western blotting was used to verify whether mTOR is involved in mediating the cell cycle arrest and apoptotic effects of compound **1t** in HCT116 cells. As evident in Figure 13, phosphorylated to unphosphorylated forms (p-mTOR/mTOR) were concentration-dependently reduced. As mTOR is downstream of AKT, Western blotting was addressed for phosphorylated to unphosphorylated forms of AKT and results unveiled a concentration-dependent decrease in p-AKT/AKT. Subsequently, checking PI3K as upstream of AKT also showed a concentration-dependent decrease in p-PI3K/PI3K. Finally, dose-dependent inhibition of the activation of the upstream MET receptor tyrosine kinase (AKA HGFR; hepatocyte growth factor receptor) without affecting the total protein expression was confirmed, as shown in Figure 13. Together, these findings suggest that the antiproliferative effects of compound **1t** are mediated by suppression of MET receptor tyrosine kinase activation, and invariably lead to down-regulation of the PI3K/AKT/mTOR signaling pathways. However, it is possible the suppression of activation of MET found might arise from other factors and signaling pathways rather than direct interaction with MET, and this might be explored in the future.

### 2.5. In Silico Docking Simulation

**Figure 14:**
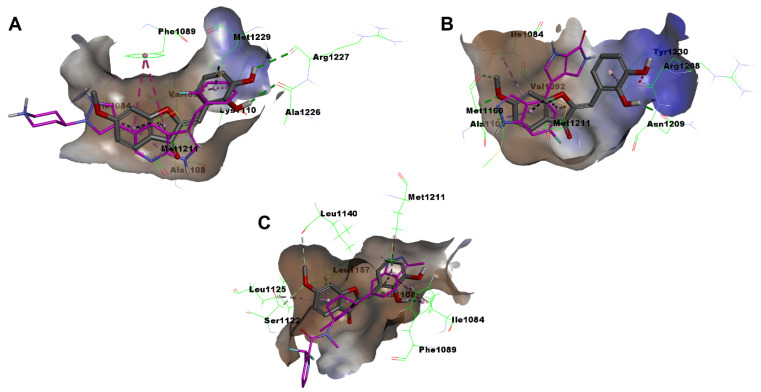
Molecular docking predicted binding modes of compound **1t** with different conformation of MET receptor tyrosine kinase: (**A**) Predicted binding mode with folded p-loop conformation (PDB: 7b41; co-crystallized ligand is pink-colored). (**B**) Predicted binding mode with extended p-loop conformation (PDB: 7b3w; co-crystallized ligand is pink-colored). (**C**) Predicted binding mode with rearranged αC helix conformation (PDB: 8an8; co-crystallized ligand is pink-colored).

The molecular target identified, MET receptor tyrosine kinase, is amongst few kinases that were recently found to possess distinct rare loop conformations [56,57,58]. These conformations are important to achieve selective inhibition. In addition to the regularly extended phosphate-binding loop (p-loop) conformation of MET receptor tyrosine kinase, researchers from the pharmaceutical industry company AstraZeneca have recently reported a rare, folded p-loop conformation of MET receptor tyrosine kinase which is collapsed or arranged into the ATP binding site that might provide a structural basis for selective targeting of MET receptor tyrosine kinase [57]. Moreover, another rearranged αC helix conformation was also reported recently by researchers from AstraZeneca to form two helices and occupy the DFG-pocket, which provides an alternative structural basis for selective targeting of MET receptor tyrosine kinase [58]. To explore which conformation might be involved in the binding of compound **1t** with MET receptor tyrosine kinase, an in silico study was conducted employing crystal structures of folded p-loop, extended p-loop, and rearranged αC helix conformations of MET receptor tyrosine kinase (protein data bank accession codes: 7b41, 7b3w, and 8an8, respectively). The results showed that compound **1t** was able to dock perfectly into the p-folded MET receptor tyrosine kinase conformation (PDB: 7b41), showing a predicted binding mode that overlayed the reported X-ray co-crystallized ligand (Figure 14A). It elicited a favorable score of −6.98019 and established a network of favorable interactions. Importantly, amongst these interactions was a crucial hydrophobic interaction with the conserved aromatic residue of the c-MET P-loop, Phe1089. In addition, two hydrogen-bonding interactions were established between the 2′,3′-dihydroxy groups of compound **1t** with Ala1226 and Arg1227 residues in the β-turn motif formed from the A-loop residues 1223–1227. Moreover, a hydrophobic interaction was formed with the catalytic residue Lys1110. On the other side, the prediction of the binding mode of compound **1t** to the extended p-loop conformation (PDB: 7b3w) revealed that the predicted binding has lower score (−6.27824) relative to the folded p-conformation and is not aligned over the co-crystalized ligand (Figure 14B). More importantly, it was unable to establish crucial interactions with Pro1158 and Met1160 residues of hinge region residues that were indispensable for binding of the co-crystallized ligand with the extended p-loop conformation. While the co-crystallized ligand in the rearranged αC helix conformation of MET receptor tyrosine kinase (PDB: 8an8) inserts between the two helices resulting from rearrangement, the prediction of the binding mode of compound **1t** unveiled its inability to insert between these two helices (Figure 14C) and, thus, it was unable to establish the crucial hydrogen bonding with the backbone of Ser1122 in the hinge connecting these helices. Together, these results might nullify the possible binding of compound **1t** to any of the extended p-loop or the rearranged αC helix conformations but suggests its binding to the folded p-loop conformations of MET receptor tyrosine kinase. In fact, compounds that inhibit the activation loop can function as a switch control and such phenomena was reported for ripretinib with KIT and PDGFRA kinases [59]. 

## 3. Materials and Methods

### 3.1. Chemistry

Synthesis and structural elucidation of compounds was reported earlier [45,60,61] and indicated in the supporting information. 

### 3.2. Biological Evaluations

#### 3.2.1. In Vitro Profiling against Human Cancer Cells Panels

Screening against the cancer cell lines was carried out according to the known standard NCI protocol [9]. 

#### 3.2.2. In Vitro Evaluation of Antiproliferative Mechanisms

Evaluation of cytotoxic mechanisms was conducted following standard protocols as described in Supplementary Materials [62,63]. 

### 3.3. In Silico Simulation Study

In silico docking study was conducted following standard protocols as described in Supplementary Materials. 

## 4. Conclusions

Nine panels consisting of diverse cancer cell lines belonging to nine cancer diseases of different origins (blood, lung, colon, CNS, skin, ovary, renal, prostate, and breast) were used to profile the anticancer activity of the natural product sulfuretin as a starting point and a library of 24 sulfuretin congeners. In contrast to the poor anticancer activity of sulfuretin at the tested concentration, compound **1t** combining the dihydroxy moieties at vicinal *ortho*- and *meta*-positions of ring-B with methoxy moiety at the *C*^6^-position of ring-A, as well as compound **1n** having *C*^6^-hydroxy moiety ring-A but possessing dimethoxy moieties at both *meta*-positions of ring-B, showed significant activities. Both compounds **1t** and **1n** were potentially active against lung, colon, brain, skin, ovarian, renal, prostate, and breast cancers. Amongst the explored compounds, the potential activities of compounds **1t** and **1n** might be associated with the structural features of compound **1t** and to a lesser extent of compound **1n**. Therefore, compound **1t** was explored for its mechanism of action in colon cancer using the HCT116 cell line. Compound **1t** was found to trigger G_0_/G_1_ cell cycle arrest and induce apoptosis through downregulating CDK4, CDK6, Cdc25A, and Cyclin D and E while increasing levels of cleaved-caspases 8 and 9 and cleaved-PARP in a dose-dependent manner. As an upstream signaling pathway involved in the regulation of the cell cycle and apoptosis in colon cancer, the impact of compound **1t** on the MET/PI3K/AKT/mTOR intracellular pathway was assessed which revealed that MET receptor tyrosine kinase and its downstreams were inhibited in a concentration-dependent manner, suggesting that MET receptor tyrosine kinase is the molecular target mediating the antiproliferative activity of compound **1t** in HCT116 colon cancer cells. The in silico study conducted to identify the conformation responsible for binding showed that compound **1t** was able to generate a binding mode with the folded p-conformation which established key interactions with crucial resides, possess a favorable energy score, and perfectly overlay with the co-crystalized crystal ligand. In contrast, the regular extended p-loop conformation or the rearranged αC helix conformation missed did not establish crucial interactions, showed lower energy scores, and did satisfactorily overlay compound **1t** with the reported co-crystalized ligands. Consequently, it was concluded that the folded p-loop conformation is the conformation responsible for the inhibition of MET receptor tyrosine kinase by compound **1t**. 

In summary, starting from the natural product sulfuretin eliciting a modest anticancer activity, compound **1t** was discovered amongst tested sulfuretin congeners as a potential anticancer agent. It was found to induce death of colon cancer cells through the triggering cell cycle arrest and apoptosis, and a mechanistic study revealed MET receptor tyrosine kinase as the molecular target mediating its anticancer activity. In silico study suggested that the folded p-loop conformation of MET receptor tyrosine kinase is the conformation responsible for binding of compound **1t**. Collectively, these findings suggest compound **1t** as a lead compound for further development of MET receptor tyrosine kinase inhibitors with potential anticancer activity. 

## Figures and Tables

**Table 1:** Sulfuretin and list of its congeners (**1a–1x**) employed in profiling anticancer activity and their literature reported inhibition of PGE_2_ production.

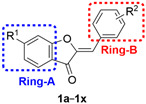							
Comp.	R^1^	R^2^	PGE_2_ IC_50_ (µM)	Comp.	R^1^	R^2^	PGE_2_ IC_50_ (µM)
**1a**	6-Hydroxy	2′-Hydroxy	1.80	**1n**	6-Hydroxy	3′,5′-Dimethoxy	1.67
**1b**	6-Hydroxy	3′-Hydroxy	9.30	**1o**	6-Hydroxy	2′,3′,4′-Trimethoxy	NR
**1c**	6-Hydroxy	4′-Hydroxy	1.06	**1p**	6-Hydroxy	4′-Methoxymethoxy	NR
**1d**	6-Hydroxy	2′,4′-Dihydroxy	18.62	**1q**	6-Methoxy	2′-Hydroxy	2.22
**1e**	6-Hydroxy	2′,5′-Dihydroxy	NR ^1^	**1r**	6-Methoxy	3′-Hydroxy	13.15
**1f**	6-Hydroxy	3′,5′-Dihydroxy	NR	**1s**	6-Methoxy	4′-Hydroxy	35.82
**1g**	6-Hydroxy	3′,4′,5′-Trihydroxy	37.62	**1t**	6-Methoxy	2′,3′-Dihydroxy	NR
**1h**	6-Hydroxy	2′-Methoxy	18.10	**1u**	6-Methoxy	2′,4′-Dihydroxy	8.80
**1i**	6-Hydroxy	3′-Methoxy	3.79	**1v**	6-Methoxy	3′,4′-Dihydroxy	4.90
**1j**	6-Hydroxy	4′-Methoxy	2.00	**1w**	6-Methoxy	2′-Methoxy	59.50
**1k**	6-Hydroxy	2′,3′-Dimethoxy	NR	**1x**	6-Methoxy	3′-Methoxy	2.50
**1l**	6-Hydroxy	2′,5′-Dimethoxy	NR	**Sulfuretin**	6-Hydroxy	3′,4′-Dihydroxy	5.90
**1m**	6-Hydroxy	3′,4′-Dimethoxy	2.90				

^1^ NR: No reported literature data.

**Table 2:** Inhibitory effects of compound **1t** on the proliferation of three human cancer cell lines.

Compound	Cell Line ^1^		
	A549	HCT116	MDAMB231
	IC_50_ (μM) ^2^	IC_50_ (μM) ^2^	IC_50_ (μM) ^2^
**1t**	19.7	**8.68**	>50

^1^ Human cancer cell lines were A549 (non-small lung cancer cell), HCT116 (colon cancer cell), and MDAMB231 (breast cancer cell). ^2^ IC_50_ results were expressed as the calculated half maximal inhibitory concentration of compound **1t** expressed in micromolar concentrations, μM.

## Data Availability

Data is contained within the article or Supplementary Materials.

## Supplementary Materials

The following supporting information can be downloaded at: https://www.mdpi.com/article/10.3390/ph16111597/s1, Tables S1–S9: detailed experimental data.
