# Supplementary material for: Repurposing Synthetic Congeners of a Natural Product Aurone Unveils a Lead Antitumor Agent Inhibiting Folded P-Loop Conformation of MET Receptor Tyrosine Kinase
Source: Pharmaceuticals (Basel). 2023 Nov 13;16(11):1597. doi: 10.3390/ph16111597 (PMC10675456; doi:10.3390/ph16111597)
Supplement: Supplementary file 1 [file pharmaceuticals-16-01597-s001.zip › pharmaceuticals-2647232-supplementary.pdf]

Supplementary Materials for

Repurposing Synthetic Congeners of a Natural Product Aurone Unveils a Lead Antitumor Agent Inhibiting Folded P-Loop Conformation of MET Receptor Tyrosine Kinase

Ahmed H.E. Hassan [1,2,†], Cai Yi Wang [3,†], Cheol Jung Lee [4], Hye Rim Jeon [4], Yeonwoo Choi [4], Suyeon Moon [4], Chae Hyeon Lee [4], Yeon Ju Kim [4], Soo Bin Cho [4], Kazem Mahmoud [5], Selwan M. El-Sayed [1], Sang Kook Lee [3,*] and Yong Sup Lee [2,4,*]

[1] Department of Medicinal Chemistry, Faculty of Pharmacy, Mansoura University, Mansoura 35516, Egypt

[2] Medicinal Chemistry Laboratory, Department of Pharmacy, College of Pharmacy, Kyung Hee University, 26 Kyungheedae-ro, Seoul 02447, Republic of Korea

[3] Natural Products Research Institute, College of Pharmacy, Seoul National University, Seoul 08826, Republic of Korea

[4] Department of Fundamental Pharmaceutical Sciences, Kyung Hee University, Seoul 02447, Republic of Korea

[5] Department of Pharmaceutical Chemistry, Faculty of Pharmacy, Egyptian Russian University, Badr City, Cairo, 11829, Egypt

*, Correspondence: kyslee@khu.ac.kr (Y.S.L.); sklee61@snu.ac.kr (S.K.L.)

† These authors contributed equally to this work.

1. Chemistry

1.1. Preparation of 6-methoxy-3-coumaranone [1]

Prepared as reported previously [1]. Yield = 58%.

^{1}H NMR (400 MHz, $CDCl_3$): δ 7.58 (d, J = 8.0 Hz, 1H), 6.66 (dd, J = 8.0, 2.0 Hz, 1H), 6.55 (d, J = 2.0 Hz, 1H), 4.63 (s, 2H), 3.88 (s, 3H, OCH_3).

1.2. Preparation of 4-(methoxymethoxy)benzaldehyde [1]

Prepared as reported previously [1]. Yield = 78%.

^{1}H NMR (400 MHz, $CDCl_3$) δ 9.91 (s, 1H), 7.89 (tt, J = 5.2, 2.6, 1.8 Hz, 2H), 7.22 (tt, J = 6.8, 2.8, 1.6 Hz, 2H), 5.78 (s, 2H), 3.42 (s, 3H).

1.3. Preparation of sulfuretin analogs [1]

Targeted compounds were prepared as reported previously [1] employing acid-catalyzed cross-aldol condensation or base-catalyzed cross-aldol condensation.

(*Z*)-6-Hydroxy-2-(2-hydroxybenzylidene)benzofuran-3(2*H*)-one (1a) [2]

Compound **1a** was prepared as reported previously [2] employing acid-catalyzed cross-aldol condensation. Yield = 75%.

^{1}H NMR (400 MHz, DMSO-d_6): δ 11.2 (brs, 1H), 10.3 (brs, 1H), 8.09 (dd, J = 6.4, 1.4 Hz, 1H), 7.61 (d, J = 8.5 Hz, 1H), 7.23 (dd, J = 6.8, 1.5 Hz, 1H'), 7.09 (s, 1H), 6.94 (m, 2H), 6.71 (dd, J = 6.5, 1.8 Hz, 1H); ^{13}C NMR (100 MHz, DMSO-d_6): δ 181.8, 186.2, 166.8, 157.6, 147.3, 131.8, 131.4, 126.3, 120.0, 119.3, 116.1, 113.4, 105.1, 99.0.

(*Z*)-6-Hydroxy-2-(3-hydroxybenzylidene)benzofuran-3(2*H*)-one (1b)

Compound **1b** was prepared as reported previously [2] employing acid-catalyzed cross-aldol condensation. Yield = 74%.

^{1}H NMR (400 MHz, DMSO-d_6): δ 7.67 (d, J = 8.4 Hz, 1H), 7.43 (d, J =1.82 Hz, 1H'), 7.38 (dd, J = 7.7, 7.3 Hz, 1H), 7.32 (dd, J = 7.9, 7.8 Hz, 1H'), 6.88 (m, 1H), 6.81 (d, J = 1.8 Hz, 1H'), 6.76 (dd, J =6.6, 1.9 Hz, 1H), 6.72 (s, 1H); ^{13}C NMR (100 MHz, DMSO-d_6): δ 181.9, 168.3, 167.0, 158.0, 147.7, 133.6, 130.6, 126.4, 122.8, 117.7,

117.5, 113.5, 113.2, 11.1, 98.9, 98.2.

(*Z*)-6-Hydroxy-2-(4-hydroxybenzylidene)benzofuran-3(2*H*)-one (1c) [3]

Compound **1c** was prepared as reported previously [3] employing acid-catalyzed cross-aldol condensation. Yield = 78%.

^{1}H NMR (400 MHz, DMSO-d_6): δ 11.1 (s, 1H), 10.1 (s, 1H), 7.83 (d, J = 8.6 Hz, 2H), 7.61 (d, J = 8.4 Hz, 1H), 6.90 (d, J = 8.6 Hz, 2H), 6.78 (d, J = 1.6 Hz, 1H), 6.73 (s, 1H), 6.72 (dd, J = 8.4, 1.6 Hz, 1H); ^{13}C NMR (400 MHz, DMSO-d_6): δ 181.7, 168.0, 166.6, 159.7, 146.2, 133.7, 126.2, 123.5, 116.5, 113.6, 113.3, 111.9, 99.0

(*Z*)-2-(2,4-Dihydroxybenzylidene)-6-hydroxybenzofuran-3(2*H*)-one (1d) [2]

Compound **1d** was prepared as reported previously [2] employing base-catalyzed cross-aldol condensation. Yield = 7% yield.

^{1}H NMR (400 MHz, MeOD-d_4): δ 10.26 (brs, 1H), 10.00 (brs, 1H), 7.94 (d, J = 9.2 Hz, 1H), 7.57 (d, J = 8.4 Hz, 1H), 7.10 (s, 1H), 6.75 (d, J = 1.8 Hz, 1H), 6.68 (dd, J = 6.4, 1.9 Hz, 1H), 6.38 (m, 2H); ^{13}C NMR (100 MHz, MeOD-d_4): δ 180.9, 167.1, 165.8, 160.8, 159.0, 145.0, 132.3, 125.4, 113.3, 112.6, 110.4, 108.2, 105.8, 102.2, 98.4.

(*Z*)-2-(2,5-Dihydroxybenzylidene)-6-hydroxybenzofuran-3(2*H*)-one (1e) [2]

Compound **1e** was prepared as reported previously [2] employing acid-catalyzed cross-aldol condensation. Yield = 7% yield).

^{1}H NMR (400 MHz, MeOD-d_4): δ 7.68 (s, 1H), 7.63 (d, J = 8.0 Hz, 1H), 7.29 (s, 1H), 6.72-6.69 (m, 4H); ^{13}C NMR (400 MHz, MeOD-d_4): δ 182.5, 167.8, 166.1, 150.2, 149.1, 146.6, 124.8, 118.8, 118.0, 115.7, 115.1, 112.7, 111.9, 106.0, 97.2.

(*Z*)-2-(3,5-Dihydroxybenzylidene)-6-hydroxybenzofuran-3(2*H*)-one (1f) [1]

Compound **1f** was prepared as reported previously [1] employing acid-catalyzed cross-aldol condensation. Yield = 7% yield.

^{1}H NMR (500 MHz, DMSO-d_6): δ 11.2 (brs, 1H), 9.47 (s, 2H), 7.59 (d, J = 8.4 Hz, 1H), 6.78 (d, J = 2.0 Hz, 2H), 6.68 (m, 2H), 6.52 (s, 1H), 6.27 (t, J = 2.2 Hz, 1H'); ^{13}C NMR (125 MHz, DMSO-d_6): δ 182.0, 168.4, 167.1, 159.1, 147.7, 133.8, 126.6, 113.6, 113.3, 111.6, 109.8, 105.0, 98.9; HRMS calcd for C15H11O5 [M+H]+ 271.0607, found 271.0623.

(*Z*)-6-Hydroxy-2-(3,4,5-trihydroxybenzylidene)benzofuran-3(2*H*)-one (1g) [1]

Compound **1g** was prepared as reported previously [1] employing acid-catalyzed cross-aldol condensation. Yield = 72%.

^{1}H NMR (400 MHz, DMSO-d_6): δ 11.15 (brs, 1H), 9.18 (brm, 2H), 7.60 (d, J = 8.4 Hz, 1H), 6.95 (s, 2H), 6.73-6.69 (m, 2H), 6.54 (s, 1H); 13C NMR (100 MHz, DMSO-d_6): δ 181.5, 167.8, 166.5, 146.5, 146.1, 136.7, 126.2, 122.6, 113.6, 113.3, 111.3, 98.7.

(*Z*)-6-Hydroxy-2-(2-methoxybenzylidene)benzofuran-3(2*H*)-one (1h) [1]

Compound **1h** was prepared as reported previously [1] employing acid-catalyzed cross-aldol condensation. Yield = 78% yield.

^{1}H NMR (400 MHz, DMSO-d_6): δ 11.19 (brs, 1H), 8.15 (dd, J = 6.3, 1.4 Hz, 1H), 7.62 (d, J = 8.4 Hz, 1H), 7.43 (m, 1H), 7.10 (m, 2H), 7.06 (s, 1H), 6.79 (d, J = 1.84 Hz, 1H), 6.72 (dd, J = 6.5, 1.8 Hz, 1H), 3.89 (s, 3H); ^{13}C NMR (100 MHz, DMSO-d_6): δ 181.3, 167.8, 166.4, 158.0, 147.3, 131.4, 130.9, 125.9, 120.8, 120.2, 113.0, 112.7, 111.4, 103.6, 98.6, 55.7.

(*Z*)-6-Hydroxy-2-(3-methoxybenzylidene)benzofuran-3(2*H*)-one (1i) [1]

Compound **1i** was prepared as reported previously [1] employing acid-catalyzed cross-aldol condensation. Yield = 92% yield.

^{1}H NMR (400 MHz, DMSO-d$_6$): δ 11.16 (brs, 1H), 7.61 (d, J = 8.4 Hz, 1H), 7.52 (d, J = 7.7 Hz, 1H), 7.47 (d, J = 2.1 Hz, 1H), 7.39 (t, J = 8.0 Hz, 1H), 6.99 (dd, J = 5.6, 2.5, 1.9 Hz, 1H), 6.79 (d, J = 1.8 Hz, 1H), 6.74 (s, 1H), 6.71 (dd, J = 6.4, 1.9 Hz, 1H), 3.80 (s, 3H); ^{13}C NMR (100 MHz, DMSO-d$_6$): δ 181.9, 168.4, 167.1, 159.8, 147.9, 133.7, 130.4, 126.4, 123.9, 116.7, 115.8, 113.6, 113.1, 110.7, 99.1, 55.6.

(*Z*)-6-Hydroxy-2-(4-methoxybenzylidene)benzofuran-3(2*H*)-one (1j) [1]

Compound **1j** was prepared as reported previously [1] employing acid-catalyzed cross-aldol condensation. Yield = 87% yield.

^{1}H NMR (400 MHz, DMSO-d_6): δ 11.17 (brs, 1H), 7.98 (d, J = 8.8 Hz, 2H), 7.68 (d, J = 8.4 Hz, 1H), 7.12 (d, J = 8.8 Hz, 2H), 6.86 (d, J = 1.8 Hz, 1H), 6.84 (s, 1H), 6.79 (dd, J = 6.5, 1.9 Hz, 1H), 3.89 (s, 3H); ^{13}C NMR (100 MHz, DMSO-$d6$): δ 181.7, 168.1, 166.7, 160.9, 146.6, 133.4, 126.2, 125.0, 115.0, 113.5, 113.3, 111.2, 99.0, 55.7.

(*Z*)-2-(2,3-Dimethoxybenzylidene)-6-hydroxybenzofuran-3(2*H*)-one (1k) [1]

Compound **1k** was prepared as reported previously [1] employing acid-catalyzed cross-aldol condensation. Yield = 97% yield.

^{1}H NMR (400 MHz, DMSO-d_6): δ 11.12 (brs, 1H), 7.80 (dd, J = 8.0, 1.6 Hz, 1H), 7.65 (d, J = 8.4 Hz, 1H), 7.24 (t, J = 8.0 Hz, 1H), 7.17 (dd, J = 8.0, 1.6 Hz, 1H), 6.98 (s, 1H), 6.79 (d, J = 1.8 Hz, 1H), 6.74 (dd, J = 8.4, 1.8 Hz, 1H), 3.86 (s, 3H), 3.83 (s, 3H); ^{13}C NMR (125 MHz, DMSOd_6): δ 182.0, 168.5, 167.2, 153.1, 152.7, 148.7, 148.5, 126.6, 125.1, 122.9, 115.1, 113.7, 113.2, 104.2, 99.2, 61.6, 56.3.

(*Z*)-2-(2,5-Dimethoxybenzylidene)-6-hydroxybenzofuran-3(2*H*)-one (1l) [1]

Compound **1l** was prepared as reported previously [1] employing acid-catalyzed cross-aldol condensation. Yield = 93% yield.

^{1}H NMR (400 MHz, DMSO-d_6): δ 11.2 (brs, 1H), 7.70 (d, J = 2.4 Hz, 1H), 7.64 (d, J = 8.4 Hz, 1H), 7.05-7.02 (m, 3H), 6.82 (d, J = 1.8 Hz, 1H), 6.74 (dd, J = 8.4, 1.8 Hz, 1H), 3.85 (s, 3H), 3.79 (s, 3H); ^{13}C NMR (100 MHz, DMSO-d_6): δ 181.9, 168.4, 167.1, 153.6, 153.1, 148.0, 126.5, 121.4, 117.0, 116.7, 113.6, 113.3, 113.0, 104.1, 99.3, 56.7, 56.0.

(*Z*)-2-(3,4-Dimethoxybenzylidene)-6-hydroxybenzofuran-3(2*H*)-one (1m) [1]

Compound **1m** was prepared as reported previously [1] employing acid-catalyzed cross-aldol condensation. Yield = 50%.

^{1}H NMR (400 MHz, DMSO-d_6): δ 11.1 (brs, 1H), 7.62-7.54 (m, 3H), 7.06 (d, J = 8.2 Hz, 1H), 6.80 (d, J = 15.0

Hz, 1H), 6.77 (s, 1H), 6.71 (d, *J* = 8.3 Hz, 1H), 3.82 (s, 6H); ^{13}C NMR (100 MHz, DMSO-d_6): δ 181.6, 168.0, 166.7, 150.8, 149.1, 146.6, 126.2, 125.5, 125.2, 114.6, 113.4, 113.0, 112.3, 111.6, 99.0, 55.9 (2C).

(*Z*)-2-(3,5-Dimethoxybenzylidene)-6-hydroxybenzofuran-3(2*H*)-one (1n) [1]

Compound **1n** was prepared as reported previously [1] employing acid-catalyzed cross-aldol condensation. Yield = 67%.

^{1}H NMR (400 MHz, Pyridine-d_5): δ 7.82 (d, *J* = 8.4 Hz, 1H), 7.29 (d, *J* = 2.1 Hz, 2H), 7.19 (s, 1H), 7.02 (dd, *J* = 5.2, 1.8 Hz, 1H), 6.93 (dd, *J* = 6.5, 1.8 Hz, 1H), 6.70 (dd, *J* = 2.1 Hz, 1H), 3.72 (s, 6H).

(*Z*)-6-Hydroxy-2-(2,3,4-trimethoxybenzylidene)benzofuran-3(2*H*)-one (1o) [1]

Compound **1o** was prepared as reported previously [1] employing acid-catalyzed cross-aldol condensation. Yield = 95%.

^{1}H NMR (500 MHz, DMSO-d_6): δ 11.16 (brs, 1H), 7.90 (d, *J* = 8.4 Hz, 1H), 7.57 (d, *J* = 8.1 Hz, 1H), 6.96 (d, *J* = 6.9 Hz, 1H), 6.86 (s, 1H), 6.73 (d, *J* = 1.9 Hz, 1H), 6.67 (dd, *J* = 8.1, 1.9 Hz, 1H), 3.84 (s, 6H), 3.74 (s, 3H); 13C NMR (125 MHz, DMSO-*d*6): δ 181.6, 168.2, 166.9, 155.7, 153.5, 147.4, 142.2, 126.9, 126.5, 118.8, 113.5 (2C), 109.2, 104.6, 99.1, 62.2, 61.0, 56.6.

(*Z*)-6-Hydroxy-2-(4-(methoxymethoxy)benzylidene)benzofuran-3(2*H*)-one (1p) [1]

Compound **1p** was prepared as reported previously [1] employing base-catalyzed cross-aldol condensation. Yield = 86%.

^{1}H NMR (400 MHz, DMSO-d_6) δ 11.16 (brs, 1H), 7.92 (d, *J* = 8.8 Hz, 2H), 7.62 (d, *J* = 8.4 Hz, 1H), 7.14 (d, *J* = 8.8 Hz, 2H), 6.79 (d, *J* = 1.8 Hz, 2H), 6.71 (dd, *J* = 8.4, 1.8 Hz, 1H), 5.27 (s, 2H), 3.37 (s, 3H) ; ^{13}C NMR (125 MHz, DMSO-d_6): δ 181.9, 168.3, 166.9, 158.4, 146.9, 133.4, 133.2, 126.4, 126.1, 117.0, 116.3, 113.5, 111.1, 99.1, 98.3, 94.2, 56.3.

(*Z*)-2-(2-Hydroxybenzylidene)-6-methoxybenzofuran-3(2*H*)-one (1q) [1]

Compound **1q** was prepared as reported previously [1] employing acid-catalyzed cross-aldol condensation. Yield = 76%.

^{1}H NMR (400 MHz, DMSO-d_6): δ 10.4 (s, 1H), 8.15 (dd, J = 8.0, 1.2 Hz, 1H), 7.70 (d, J = 8.4 Hz, 1H), 7.31 (td, J = 8.0, 1.2 Hz, 1H), 7.17 (d, J = 2.0 Hz, 1H), 7.16 (s, 1H), 6.98−6.93 (m, 2H), 6.87 (dd, J = 8.4, 2.0 Hz, 1H), 3.93 (s, 3H); ^{13}C NMR (100 MHz, DMSO-d_6): δ 181.6, 167.8, 167.2, 157.2, 146.8, 131.5, 131.0, 125.4, 119.6, 118.7, 115.8, 114.0, 112.6, 105.2, 97.1, 56.4.

(*Z*)-2-(3-Hydroxybenzylidene)-6-methoxybenzofuran-3(2*H*)-one (1r) [1]

Compound **1r** was prepared as reported previously [1] employing acid-catalyzed cross-aldol condensation. Yield = 27%.

^{1}H NMR (400 MHz, DMSO-d_6): δ 9.73 (s, 1H), 7.72 (d, J = 8.4 Hz, 1H), 7.41−7.40 (m, 2H) 7.32 (t, J = 8.0 Hz, 1H), 7.15 (d, J = 2.0, 1H), 6.88−6.86 (m, 2H), 6.76 (s, 1H), 3.94 (s, 3H); ^{13}C NMR (100 MHz, DMSO-d_6): δ 182.2, 168.5, 167.9, 158.1, 147.6, 133.5, 130.4, 126.0, 122.9, 118.0, 117.7, 114.3, 113.2, 111.7, 97.6, 56.9.

(*Z*)-2-(4-Hydroxybenzylidene)-6-methoxybenzofuran-3(2*H*)-one (1s) [1]

Compound **1s** was prepared as reported previously [1] employing acid-catalyzed cross-aldol condensation. Yield = 39%.

^{1}H NMR (400 MHz, DMSO-d_6): δ 10.2 (brs, 1H), 7.87 (d, J = 8.8 Hz, 2H), 7.69 (d, J = 8.4 Hz, 1H), 7.16 (d, J = 2.0 Hz, 1H), 6.91 (d, J = 8.8 Hz, 2H), 6.87 (dd, J = 8.4, 2.0 Hz, 1H), 6.80 (s, 1H), 3.93 (s, 3H); ^{13}C NMR (100 MHz, DMSO-d_6): δ 181.4, 167.5, 167.0, 159.4, 145.6, 133.4 (2C), 125.2, 122.9, 116.0 (2C), 114.2, 112.4, 112.0, 97.0, 56.3.

(*Z*)-2-(2,3-Dihydroxybenzylidene)-6-methoxybenzofuran-3(2*H*)-one (1t) [1]

Compound **1t** was prepared as reported previously [1] employing acid-catalyzed cross-aldol condensation. Yield = 26%.

^{1}H NMR (500 MHz, DMSO-d_6): δ 7.64 (d, J = 8.6 Hz, 1H), 7.57 (dd, J = 8.0, 1.5 Hz, 1H), 7.14 (s, 1H), 7.11 (d, J = 2.2 Hz, 1H), 6.82−6.79 (m, 2H), 6.71 (t, J = 7.9 Hz, 1H), 3.88 (s, 3H) , 3.12 (s, 1H), , 2.49 (s, 1H); ^{13}C NMR (125 MHz, DMSO-*d6*): δ 182.1, 168.3, 167.7, 147.2, 146.8, 146.1, 125.9, 121.8, 119.9, 119.8, 117.4, 114.6, 113.1, 106.5, 97.7, 40.9; HRMS calcd for C16H13O5 $[M+H]^+$ 285.0763, found 285.0764.

(*Z*)-2-(2,4-Dihydroxybenzylidene)-6-methoxybenzofuran-3(2*H*)-one (1u) [1]

Compound **1u** was prepared as reported previously [1] employing acid-catalyzed cross-aldol condensation. Yield = 12%.

^{1}H NMR (400 MHz, DMSO-d_6): δ 10.40 (s, 1H), 10.10 (s, 1H), 8.02 (d, J = 8.4 Hz, 1H), 7.67 (d, J = 8.8 Hz, 1H), 7.14 (d, J = 2.4 Hz, 1H), 7.10 (s, 1H), 6.86 (dd, J = 8.8, 2.4 Hz, 1H), 6.42–6.38 (m, 2H), 3.90 (s, 3H); ^{13}C NMR (100 MHz, DMSO-d_6): δ 181.1, 167.2, 166.7, 161.0, 159.3, 145.0, 132.5, 125.0, 114.5, 112.3, 110.5, 108.3, 106.5, 102.3, 97.0, 56.3.

(*Z*)-2-(3,4-Dihydroxybenzylidene)-6-methoxybenzofuran-3(2*H*)-one (1v) [1]

Compound **1v** was prepared as reported previously [1] employing acid-catalyzed cross-aldol condensation. Yield = 58%.

^{1}H NMR (400 MHz, methanol-d_4) δ 7.68 (dd, J = 8.8, 4.4 Hz, 1H), 7.52 (s, 1H), 7.29 (dd, J = 8.4, 2.4 Hz, 1H), 6.95 (d, J = 2.4 Hz, 1H), 6.86–6.82 (m, 2H), 6.73 (d, J = 4.4 Hz, 1H), 3.96 (s, 3H); ^{13}C NMR (100 MHz, DMSO-d_6) δ 181.3, 167.4, 166.9 (2C), 148.2, 145.5, 125.2, 124.6, 123.3, 118.2, 116.0, 114.3, 112.5, 112.4, 96.9, 56.3.

(*Z*)-6-Methoxy-2-(2-methoxybenzylidene)benzofuran-3(2*H*)-one (1w) [1]

Compound **1w** was prepared as reported previously [1] employing acid-catalyzed cross-aldol condensation. Yield = 69%.

^{1}H NMR (400 MHz, $CDCl_3$) δ 8.27 (dd, J = 8.0, 2.0 Hz, 1H), 7.72 (d, J = 8.4 Hz, 1H), 7.40 (s, 1H), 7.38 (td, J = 8.0, 2.0 Hz, 1H), 7.07 (t, J = 8.0 Hz, 1H), 6.94 (d, J = 8.0 Hz, 1H), 6.77–6.73 (m, 2H), 3.92 (s, 3H), 3.90 (s, 3H); ^{13}C NMR (100 MHz, $CDCl_3$) δ 183.0, 168.4, 167.2, 158.7, 147.9, 131.8, 131.1, 125.8, 121.5, 120.8, 115.1, 112.0, 110.8, 106.1, 96.6, 56.0, 55.6.

(*Z*)-6-Methoxy-2-(3-methoxybenzylidene)benzofuran-3(2*H*)-one (1x) [1]

Compound **1x** was prepared as reported previously [1] employing acid-catalyzed cross-aldol condensation. Yield = 36%.

^{1}H NMR (400 MHz, $CDCl_3$) δ 7.72 (d, J = 8.0 Hz, 1H), 7.48–7.46 (m, 2H), 7.38 (t, J = 8.0 Hz, 1H), 6.96 (m, J =

8.0, 2.0 Hz, 1H), 6.79–6.75 (m, 3H), 3.93 (s, 3H), 3.88 (s, 3H); ^{13}C NMR (100 MHz, $CDCl_3$) δ 183.0, 168.6, 167.5, 159.8, 148.0, 133.7, 129.8, 125.9, 124.1, 116.5, 115.4, 114.9, 112.2, 111.7, 96.7, 56.1, 55.4.

2. *In vitro* evaluation of antiproliferative mechanisms

General Experimental Procedures. General Experimental Procedures. Roswell Park Memorial Institute (RPMI) 1640 medium, Dulbecco Modified Eagle Medium (DMEM) High glucose, Eagle Minimum Essential Medium (EMEM), fetal bovine serum (FBS), antibiotic−antimycotic solution (100×), trypsin−EDTA solution (1×), and phosphate-buffered saline (PBS; 1×) were purchased from HyClone Laboratories, Inc. (South Logan, UT, USA). Laemmli sample buffer (2×) and 2-mercaptomethanol were purchased from Bio-Rad Laboratories, Inc. (Hercules, CA, USA). Dimethyl sulfoxide (DMSO), bicinchoninic acid (BCA), copper(II) sulfate solution, bovine serum albumin (BSA), trichloroacetic acid (TCA), SRB, ribonuclease A (RNase A), and paclitaxel were purchased from Sigma-Aldrich (St. Louis, MO, USA). Anti-Cleaved PARP, anti-Caspas 8, anti-Cleaved Caspas 8, anti-Caspas 9, anti-Cleaved Caspas 9, anti-CDK4, anti-CDK6 and anti-Cdc25C were purchased from Cell Signaling Technology (Danvers, MA, USA). Anti-PARP, anti-Cyclin D1, anti-Cyclin E was purchased from BD Biosciences (Franklin Lakes, NJ, USA). Anti-β-actin were purchased from Santa Cruz Technology (Santa Cruz, CA, USA).

Cell Culture. HCT116 cells (Human colon cancer), MDA-MB-231 cells (Human breast cancer) and A549 cells (Human lung cancer) were purchased from ATCC (Manassas, VA, USA). HCT116 cells were cultured in RPMI medium, which added with 10% FBS and 1% antibiotics−antimycotics (AA). Cells were Suspended in DMEM medium which added with 10% FBS and 1% antibiotics−antimycotics (AA) (PSF; 100 units/mL penicillin G sodium, 100 μg/mL streptomycin, and 250 ng/mL amphotericin B). Cells were cultured at 37 °C in 96 well plates under a humidified atmosphere containing 5% CO_2.

Cell Viability Assay. After 72 h of sample treatment, the cell viability was measured by using the sulforhodamine B (SRB) assay as described previously [4]. Briefly, after incubated with different concentrations of compounds for 72 h, the cells were fixed with 10% trichloroacetic acid (TCA) and stained with SRB solution in 0.1% acetic acids. The stained proteins precipitate was dissolved in 10 mM Tris solution at pH 10 and measured by a Versamax ELISA machine at 515 nm.

Western blotting. HCT116 cells (1×10^5 cells/mL in a 60 mm dish) were cultured with the various concentrations of compounds for 24h, 48h. The cells were collected and lysed in 2×sample loading buffer (250 mM Tris-HCl pH 6.8, 4% SDS, 10% glycerol, 0.006% bromophenol blue, 2% β-mercaptoethanol, 50 mM sodium fluoride, and 5 mM sodium orthovanadate). Run the protein samples with the marker on 6–12% SDS-PAGE gel and transferred onto PVDF membranes (Millipore, Bedford, MA, USA). The membranes were blocked with 5% BSA in Tris-buffered saline containing 0.1% Tween-20 (TBST) for 30 min at room temperature, and then incubated with primary antibodies in 2.5% BSA in TBST overnight at 4 °C on a shaker. After the membranes were washed three times with TBST, they were incubated with the secondary antibodies (HRP) (Younginfrontier, Seoul, Korea) diluted in TBST for 2 h at room temperature. And the membranes were further exposed to enhanced chemiluminescence (ECL) solution (Intron, Daejon, Korea) after washing with TBST. The chemiluminescence signals were measured by using LAS-4000 (Fuji Film Corp., Tokyo, Japan).

Cell Cycle Analysis. The above cultured cells were starved in serum-free medium for 8h before the compound sample treatment. After treatment, cells were collected using trypsin, washed with PBS, and fixed with 70% ethanol. The cell lines were dyed with propidium iodide (PI) mixed with RNase, and flow cytometry analysis was performed as described previously [5].

Annexin V-FITC/PI Staining. After the cell lines were treated with compounds for 48 h, they were collected, washed with cold PBS, and resuspended in 1× binding buffer. Then each set of them were dyed with annexin V-FITC and PI and incubated at ambient temperature for 15 min. the analysis cell apoptosis was performed after Flow cytometry assay.

References

[1] A.H.E. Hassan, T.N. Phan, Y. Choi, S. Moon, J.H. No, Y.S. Lee, Design, Rational Repurposing, Synthesis, In Vitro Evaluation, Homology Modeling and In Silico Study of Sulfuretin Analogs as Potential Antileishmanial Hit Compounds, Pharmaceuticals, 15 (2022) 1058.

[2] A.H.E. Hassan, H.J. Kim, M.S. Gee, J.-H. Park, H.R. Jeon, C.J. Lee, Y. Choi, S. Moon, D. Lee, J.K. Lee, K.D. Park, Y.S. Lee, Positional scanning of natural product hispidol's ring-B: discovery of highly selective human monoamine oxidase-B inhibitor analogues downregulating neuroinflammation for management of neurodegenerative diseases, J. Enzyme Inhib. Med. Chem., 37 (2022) 768-780.

[3] S.Y. Shin, M.C. Shin, J.-S. Shin, K.-T. Lee, Y.S. Lee, Synthesis of aurones and their inhibitory effects on nitric oxide and PGE2 productions in LPS-induced RAW 264.7 cells, Bioorg. Med. Chem. Lett., 21 (2011) 4520-4523.

[4] W.S. Byun, M. Jin, J. Yu, W.K. Kim, J. Song, H.-J. Chung, L.S. Jeong, S.K. Lee, A novel selenonucleoside suppresses tumor growth by targeting Skp2 degradation in paclitaxel-resistant prostate cancer, Biochem. Pharmacol., 158 (2018) 84-94.

[5] C. Jung, J.-Y. Hong, S.Y. Bae, S.S. Kang, H.J. Park, S.K. Lee, Antitumor Activity of Americanin A Isolated from the Seeds of Phytolacca americana by Regulating the ATM/ATR Signaling Pathway and the Skp2–p27 Axis in Human Colon Cancer Cells, J. Nat. Prod., 78 (2015) 2983-2993.

Table S1. % Growth inhibition of diverse blood cancer cell lines triggered by 10 µM concentrations of sulfuretin and its prepared analogs (**1a–x**).

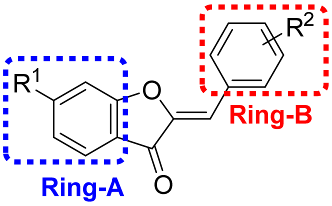

1a–x

Comp	R¹	R²	CCRFCEM	MOLT4	HL60(TB)	K562	RPMI8226
**1a**	6-Hydoxy	2'-Hydoxy	0.34	4.13	NI	2.64	NI
**1b**	6-Hydoxy	3'-Hydoxy	1.34	NI	NI	5.19	NI
**1c**	6-Hydoxy	4'-Hydoxy	3.08	NI	NI	3.43	NI
**1d**	6-Hydoxy	2',4'-Dihydoxy	NI	NI	NI	NI	NI
**1e**	6-Hydoxy	2',5'-Dihydoxy	NI	NI	0.99	1.02	NI
**1f**	6-Hydoxy	3',5'-Dihydoxy	3.81	NI	5.88	10.38	NI
**1g**	6-Hydoxy	3',4',5'-Trihydoxy	NI	NI	NI	0.65	NI
**1h**	6-Hydoxy	2'-Methoxy	NI	NI	NI	1.41	NI
**1i**	6-Hydoxy	3'-Methoxy	NI	NI	0.53	1.15	NI
**1j**	6-Hydoxy	4'-Methoxy	NI	NI	1.40	3.23	NI
**1k**	6-Hydoxy	2',3'-Dimethoxy	NI	NI	2.89	4.56	NI
**1l**	6-Hydoxy	2',5'-Dimethoxy	3.86	NI	4.32	6.78	7.77
**1m**	6-Hydoxy	3',4'-Dimethoxy	3.92	11.07	30.69	9.71	1.05
**1n**	6-Hydoxy	3',5'-Dimethoxy	26.19	15.78	54.49	30.19	12.37
**1o**	6-Hydoxy	2',3',4'-Trimethoxy	NI	1.92	30.27	0.77	0.35
**1p**	6-Hydoxy	4'-Methoxymethoxy	NI	1.12	0.76	1.71	NI
**1q**	6-Methoxy	2'-Hydoxy	NI	3.36	6.14	6.88	NI
**1r**	6-Methoxy	3'-Hydoxy	4.88	NI	2.22	3.50	7.88
**1s**	6-Methoxy	4'-Hydoxy	32.35	NI	9.18	10.69	NI
**1t**	6-Methoxy	2',3'-Dihydoxy	72.84	57.69	37.53	14.63	32.70
**1u**	6-Methoxy	2',4'-Dihydoxy	NI	NI	NI	0.68	NI
**1v**	6-Methoxy	3',4'-Dihydoxy	47.35	35.26	34.04	7.29	22.21
**1w**	6-Methoxy	2'-Methoxy	NI	0.96	8.51	13.64	NI
**1x**	6-Methoxy	3'-Methoxy	NI	NI	NI	NI	NI
**Sulfuretin**	6-Hydoxy	3',4'-Dihydoxy	17.34	11.66	NI	6.33	2.04

Table S2. % Growth inhibition of diverse non-small cell lung cancer cell lines triggered by 10 µM concentrations of sulfuretin and its prepared analogs (**1a**−**x**).

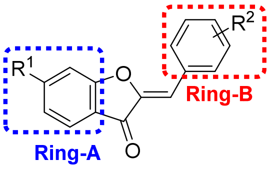

1a–x

Comp	R^1	R^2	A549	EKVX	HOP62	H23	H322M	HOP92	H460
**1a**	6-Hydoxy	2'-Hydoxy	NI	2.51	NI	NI	2.14	NI	NI
**1b**	6-Hydoxy	3'-Hydoxy	NI	5.76	NI	NI	1.06	9.70	NI
**1c**	6-Hydoxy	4'-Hydoxy	0.70	NI	NI	NI	6.56	NI	NI
**1d**	6-Hydoxy	2',4'-Dihydoxy	NI	2.99	0.03	NI	NI	4.19	NI
**1e**	6-Hydoxy	2',5'-Dihydoxy	NI	3.27	NI	NI	NI	NI	NI
**1f**	6-Hydoxy	3',5'-Dihydoxy	NI	NI	NI	NI	NI	NI	NI
**1g**	6-Hydoxy	3',4',5'-Trihydoxy	0.35	4.54	NI	NI	NI	NI	NI
**1h**	6-Hydoxy	2'-Methoxy	NI	4.15	NI	NI	NI	NI	NI
**1i**	6-Hydoxy	3'-Methoxy	5.92	7.66	1.44	1.30	NI	NI	NI
**1j**	6-Hydoxy	4'-Methoxy	0.07	6.51	NI	NI	NI	NI	NI
**1k**	6-Hydoxy	2',3'-Dimethoxy	NI	0.61	NI	NI	NI	NI	NI
**1l**	6-Hydoxy	2',5'-Dimethoxy	1.23	8.62	NI	3.91	7.43	1.80	8.36
**1m**	6-Hydoxy	3',4'-Dimethoxy	NI	NI	NI	NI	NI	NI	NI
**1n**	6-Hydoxy	3',5'-Dimethoxy	27.03	8.86	4.18	9.81	29.10	2.70	37.75
**1o**	6-Hydoxy	2',3',4'-Trimethoxy	NI	NI	NI	NI	NI	NI	NI
**1p**	6-Hydoxy	4'-Methoxymethoxy	NI	4.42	NI	2.46	4.35	NI	NI
**1q**	6-Methoxy	2'-Hydoxy	NI	3.96	3.92	7.64	2.05	7.15	NI
**1r**	6-Methoxy	3'-Hydoxy	NI	7.71	2.41	2.32	4.52	5.90	NI
**1s**	6-Methoxy	4'-Hydoxy	6.55	4.62	NI	0.06	5.50	NI	0.21
**1t**	6-Methoxy	2',3'-Dihydoxy	33.72	17.87	10.96	29.89	27.62	4.63	38.23
**1u**	6-Methoxy	2',4'-Dihydoxy	2.89	NI	NI	NI	9.05	NI	NI
**1v**	6-Methoxy	3',4'-Dihydoxy	8.48	NI	NI	NI	10.51	NI	5.90
**1w**	6-Methoxy	2'-Methoxy	8.95	NI	NI	NI	4.57	8.80	NI
**1x**	6-Methoxy	3'-Methoxy	NI	NI	NI	NI	NI	NI	NI
**Sulfuretin**	6-Hydoxy	3',4'-Dihydoxy	NI	NI	NI	NI	0.93	NI	NI

Table S3. % Growth inhibition of diverse colorectal cancer cell lines triggered by 10 μM concentrations of sulfuretin and its prepared analogs (**1a**–**x**).

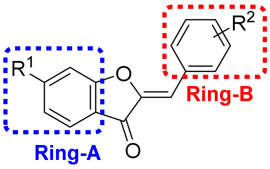

1a–x

Comp	R¹	R²	HCC2998	HCT116	HCT15	KM12	SW620
**1a**	6-Hydoxy	2'-Hydoxy	NI	NI	NI	NI	NI
**1b**	6-Hydoxy	3'-Hydoxy	NI	NI	7.28	NI	NI
**1c**	6-Hydoxy	4'-Hydoxy	NI	NI	6.80	NI	NI
**1d**	6-Hydoxy	2',4'-Dihydoxy	NI	NI	NI	NI	NI
**1e**	6-Hydoxy	2',5'-Dihydoxy	NI	3.67	NI	NI	NI
**1f**	6-Hydoxy	3',5'-Dihydoxy	NI	15.40	NI	NI	NI
**1g**	6-Hydoxy	3',4',5'-Trihydoxy	NI	16.80	NI	NI	NI
**1h**	6-Hydoxy	2'-Methoxy	NI	NI	1.10	NI	NI
**1i**	6-Hydoxy	3'-Methoxy	NI	1.04	6.73	NI	NI
**1j**	6-Hydoxy	4'-Methoxy	NI	15.48	0.83	NI	NI
**1k**	6-Hydoxy	2',3'-Dimethoxy	NI	0.76	NI	NI	NI
**1l**	6-Hydoxy	2',5'-Dimethoxy	0.70	20.85	24.96	NI	7.80
**1m**	6-Hydoxy	3',4'-Dimethoxy	NI	NI	NI	3.15	NI
**1n**	6-Hydoxy	3',5'-Dimethoxy	NI	40.81	51.76	6.80	25.32
**1o**	6-Hydoxy	2',3',4'-Trimethoxy	NI	NI	NI	NI	NI
**1p**	6-Hydoxy	4'-Methoxymethoxy	NI	NI	1.66	NI	NI
**1q**	6-Methoxy	2'-Hydoxy	NI	6.96	0.36	NI	NI
**1r**	6-Methoxy	3'-Hydoxy	NI	1.35	5.01	NI	2.27
**1s**	6-Methoxy	4'-Hydoxy	0.55	NI	3.12	NI	NI
**1t**	6-Methoxy	2',3'-Dihydoxy	21.28	45.53	69.49	33.70	39.95
**1u**	6-Methoxy	2',4'-Dihydoxy	NI	2.93	4.59	9.47	NI
**1v**	6-Methoxy	3',4'-Dihydoxy	NI	11.03	24.00	22.08	4.78
**1w**	6-Methoxy	2'-Methoxy	NI	9.66	8.51	13.52	10.61
**1x**	6-Methoxy	3'-Methoxy	NI	NI	0.22	NI	NI
**Sulfuretin**	6-Hydoxy	3',4'-Dihydoxy	NI	NI	10.67	9.49	NI

Table S4. % Growth inhibition of diverse brain cancer cell lines triggered by 10 µM concentrations of sulfuretin and its prepared analogs (**1a–x**).

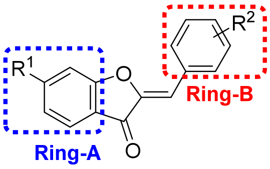

1a–x

Comp	R¹	R²	SF268	SNB75	SF295	SF539	SNB19	U251
**1a**	6-Hydoxy	2'-Hydoxy	NI	15.81	3.54	NI	2.81	NI
**1b**	6-Hydoxy	3'-Hydoxy	NI	24.83	5.38	4.56	3.50	NI
**1c**	6-Hydoxy	4'-Hydoxy	NI	15.49	1.53	11.94	12.88	3.02
**1d**	6-Hydoxy	2',4'-Dihydoxy	NI	1.32	NI	NI	NI	NI
**1e**	6-Hydoxy	2',5'-Dihydoxy	NI	NI	NI	NI	NI	NI
**1f**	6-Hydoxy	3',5'-Dihydoxy	3.63	1.99	NI	NI	NI	1.71
**1g**	6-Hydoxy	3',4',5'-Trihydoxy	NI	1.20	NI	NI	NI	NI
**1h**	6-Hydoxy	2'-Methoxy	NI	NI	NI	NI	NI	NI
**1i**	6-Hydoxy	3'-Methoxy	0.75	NI	1.02	NI	NI	5.99
**1j**	6-Hydoxy	4'-Methoxy	NI	NI	1.62	2.33	1.24	3.16
**1k**	6-Hydoxy	2',3'-Dimethoxy	NI	NI	NI	NI	NI	NI
**1l**	6-Hydoxy	2',5'-Dimethoxy	12.68	11.38	1.43	4.57	4.47	10.54
**1m**	6-Hydoxy	3',4'-Dimethoxy	11.64	13.83	1.67	3.87	9.83	2.40
**1n**	6-Hydoxy	3',5'-Dimethoxy	30.13	45.46	10.11	16.36	11.29	35.13
**1o**	6-Hydoxy	2',3',4'-Trimethoxy	NI	NI	NI	NI	4.52	NI
**1p**	6-Hydoxy	4'-Methoxymethoxy	2.23	7.33	7.70	NI	19.12	14.28
**1q**	6-Methoxy	2'-Hydoxy	3.91	0.43	NI	NI	5.45	NI
**1r**	6-Methoxy	3'-Hydoxy	4.36	8.09	1.95	4.93	2.87	0.84
**1s**	6-Methoxy	4'-Hydoxy	12.38	15.19	NI	7.49	5.00	6.65
**1t**	6-Methoxy	2',3'-Dihydoxy	47.38	37.49	6.23	41.46	15.10	39.65
**1u**	6-Methoxy	2',4'-Dihydoxy	10.53	16.25	NI	NI	4.53	3.01
**1v**	6-Methoxy	3',4'-Dihydoxy	30.38	29.82	NI	20.95	8.36	16.06
**1w**	6-Methoxy	2'-Methoxy	7.56	17.41	6.27	2.43	0.84	17.67
**1x**	6-Methoxy	3'-Methoxy	4.80	15.83	2.88	NI	0.30	NI
**Sulfuretin**	6-Hydoxy	3',4'-Dihydoxy	5.36	19.89	NI	21.03	5.68	4.36

Table S5. % Growth inhibition of diverse melanoma cancer cell lines triggered by 10 µM concentrations of sulfuretin and its prepared analogs (**1a–x**).

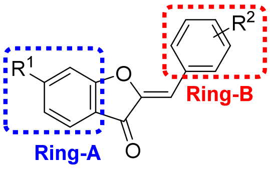

1a–x

Comp	R¹	R²	LOXIMVI	M14	MDAMB435	MALME3M	SKMEL28	SKMEL5	UACC257	UACC62
**1a**	6-Hydoxy	2'-Hydoxy	NI	NI	NI	NI	NI	1.25	NI	5.05
**1b**	6-Hydoxy	3'-Hydoxy	NI	NI	NI	3.19	NI	5.14	NI	3.09
**1c**	6-Hydoxy	4'-Hydoxy	NI	NI	1.98	NI	NI	0.62	NI	6.68
**1d**	6-Hydoxy	2',4'-Dihydoxy	NI	NI	NI	3.51	NI	0.93	0.00	0.65
**1e**	6-Hydoxy	2',5'-Dihydoxy	4.67	NI	0.90	NI	NI	2.47	NI	NI
**1f**	6-Hydoxy	3',5'-Dihydoxy	6.28	6.94	NI	6.27	NI	1.66	NI	NI
**1g**	6-Hydoxy	3',4',5'-Trihydoxy	10.36	4.35	NI	NI	NI	3.91	NI	NI
**1h**	6-Hydoxy	2'-Methoxy	NI	NI	0.11	5.85	NI	1.62	NI	NI
**1i**	6-Hydoxy	3'-Methoxy	4.26	4.25	4.70	5.34	NI	0.52	NI	NI
**1j**	6-Hydoxy	4'-Methoxy	NI	12.89	1.64	5.96	NI	0.31	NI	0.84
**1k**	6-Hydoxy	2',3'-Dimethoxy	NI	5.56	NI	5.19	NI	2.41	NI	NI
**1l**	6-Hydoxy	2',5'-Dimethoxy	11.44	26.80	17.29	23.96	11.49	7.61	NI	11.00
**1m**	6-Hydoxy	3',4'-Dimethoxy	NI	NI	2.36	NI	NI	3.20	NI	1.84
**1n**	6-Hydoxy	3',5'-Dimethoxy	37.54	46.55	26.59	36.44	13.35	26.94	20.47	30.02
**1o**	6-Hydoxy	2',3',4'-Trimethoxy	NI	NI	2.43	3.63	NI	5.81	NI	NI
**1p**	6-Hydoxy	4'-Methoxymethoxy	NI	NI	NI	2.55	15.00	8.18	NI	9.37
**1q**	6-Methoxy	2'-Hydoxy	2.15	3.96	NI	4.22	NI	1.18	NI	6.57
**1r**	6-Methoxy	3'-Hydoxy	2.35	NI	1.66	NI	NI	0.32	NI	7.73
**1s**	6-Methoxy	4'-Hydoxy	4.35	NI	12.63	2.65	NI	NI	1.83	0.66
**1t**	6-Methoxy	2',3'-Dihydoxy	53.65	9.56	25.87	25.35	17.81	28.60	0.23	27.84
**1u**	6-Methoxy	2',4'-Dihydoxy	NI	3.53	29.88	7.72	NI	NI	NI	6.57
**1v**	6-Methoxy	3',4'-Dihydoxy	12.50	NI	8.54	4.17	4.00	0.36	NI	7.38
**1w**	6-Methoxy	2'-Methoxy	NI	4.48	27.82	8.71	0.45	3.19	3.82	15.46
**1x**	6-Methoxy	3'-Methoxy	NI	NI	3.09	NI	0.46	NI	NI	8.83
**Sulfuretin**	6-Hydoxy	3',4'-Dihydoxy	NI	NI	4.57	NI	NI	7.52	NI	5.21

Table S6. % Growth inhibition of diverse ovarian cancer cell lines triggered by 10 µM concentrations of sulfuretin and its prepared analogs (**1a–x**).

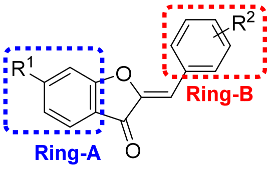

1a–x

Comp	R¹	R²	IGROV1	OVCAR3	OVCAR4	OVCAR5	OVCAR8	ADRRES
**1a**	6-Hydoxy	2'-Hydoxy	NI	NI	NI	NI	NI	NI
**1b**	6-Hydoxy	3'-Hydoxy	NI	NI	1.81	NI	NI	NI
**1c**	6-Hydoxy	4'-Hydoxy	NI	NI	NI	NI	NI	NI
**1d**	6-Hydoxy	2',4'-Dihydoxy	NI	NI	NI	NI	NI	NI
**1e**	6-Hydoxy	2',5'-Dihydoxy	NI	NI	NI	NI	NI	NI
**1f**	6-Hydoxy	3',5'-Dihydoxy	NI	NI	NI	NI	1.12	NI
**1g**	6-Hydoxy	3',4',5'-Trihydoxy	NI	NI	NI	NI	NI	NI
**1h**	6-Hydoxy	2'-Methoxy	NI	NI	NI	NI	NI	NI
**1i**	6-Hydoxy	3'-Methoxy	2.99	NI	6.04	NI	NI	1.15
**1j**	6-Hydoxy	4'-Methoxy	NI	NI	NI	NI	NI	-6.12
**1k**	6-Hydoxy	2',3'-Dimethoxy	NI	NI	NI	NI	NI	NI
**1l**	6-Hydoxy	2',5'-Dimethoxy	10.73	4.23	13.31	NI	14.42	7.10
**1m**	6-Hydoxy	3',4'-Dimethoxy	NI	NI	NI	NI	2.79	NI
**1n**	6-Hydoxy	3',5'-Dimethoxy	43.54	19.41	28.79	5.35	25.20	6.80
**1o**	6-Hydoxy	2',3',4'-Trimethoxy	NI	NI	NI	NI	NI	NI
**1p**	6-Hydoxy	4'-Methoxymethoxy	0.72	NI	NI	NI	NI	1.50
**1q**	6-Methoxy	2'-Hydoxy	NI	NI	11.58	NI	1.34	3.75
**1r**	6-Methoxy	3'-Hydoxy	2.45	NI	14.94	NI	3.53	NI
**1s**	6-Methoxy	4'-Hydoxy	0.27	NI	5.24	NI	8.97	NI
**1t**	6-Methoxy	2',3'-Dihydoxy	62.54	76.89	46.14	21.03	27.81	39.87
**1u**	6-Methoxy	2',4'-Dihydoxy	8.62	7.99	0.82	NI	0.73	1.54
**1v**	6-Methoxy	3',4'-Dihydoxy	23.35	16.75	18.63	5.13	3.57	5.72
**1w**	6-Methoxy	2'-Methoxy	4.40	3.12	4.53	3.20	1.78	NI
**1x**	6-Methoxy	3'-Methoxy	NI	NI	4.65	NI	NI	0.79
**Sulfuretin**	6-Hydoxy	3',4'-Dihydoxy	NI	0.96	4.46	NI	NI	NI

Table S7. % Growth inhibition of diverse renal cancer cell lines triggered by 10 µM concentrations of sulfuretin and its prepared analogs (**1a–x**).

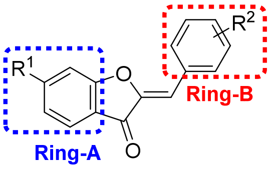

1a–x

Comp	R¹	R²	786O	CAKI1	ACHN	RXF393	SN12C	UO31
**1a**	6-Hydoxy	2'-Hydoxy	6.35	4.77	NI	NI	NI	NI
**1b**	6-Hydoxy	3'-Hydoxy	18.19	4.38	NI	NI	1.16	NI
**1c**	6-Hydoxy	4'-Hydoxy	9.49	NI	NI	NI	3.56	NI
**1d**	6-Hydoxy	2',4'-Dihydoxy	3.42	3.75	1.79	NI	1.01	13.36
**1e**	6-Hydoxy	2',5'-Dihydoxy	0.31	0.59	NI	NI	NI	1.35
**1f**	6-Hydoxy	3',5'-Dihydoxy	0.28	0.99	NI	NI	0.53	3.98
**1g**	6-Hydoxy	3',4',5'-Trihydoxy	0.93	NI	NI	NI	NI	7.89
**1h**	6-Hydoxy	2'-Methoxy	0.02	5.72	NI	NI	1.80	10.71
**1i**	6-Hydoxy	3'-Methoxy	12.66	23.69	NI	NI	NI	18.70
**1j**	6-Hydoxy	4'-Methoxy	3.59	9.11	NI	NI	NI	14.45
**1k**	6-Hydoxy	2',3'-Dimethoxy	NI	NI	NI	NI	NI	4.43
**1l**	6-Hydoxy	2',5'-Dimethoxy	10.54	6.33	31.67	19.47	14.21	39.71
**1m**	6-Hydoxy	3',4'-Dimethoxy	NI	12.69	NI	NI	5.60	8.55
**1n**	6-Hydoxy	3',5'-Dimethoxy	23.64	65.63	45.39	38.87	23.38	53.08
**1o**	6-Hydoxy	2',3',4'-Trimethoxy	NI	3.71	NI	NI	NI	11.33
**1p**	6-Hydoxy	4'-Methoxymethoxy	10.90	1.41	NI	NI	11.54	0.32
**1q**	6-Methoxy	2'-Hydoxy	11.33	1.80	NI	NI	5.74	21.90
**1r**	6-Methoxy	3'-Hydoxy	5.73	7.23	NI	NI	3.58	21.38
**1s**	6-Methoxy	4'-Hydoxy	8.21	9.26	NI	NI	2.02	23.43
**1t**	6-Methoxy	2',3'-Dihydoxy	19.63	56.79	33.79	27.15	42.29	69.47
**1u**	6-Methoxy	2',4'-Dihydoxy	5.14	5.78	NI	NI	6.70	11.41
**1v**	6-Methoxy	3',4'-Dihydoxy	1.07	27.20	NI	NI	22.35	50.03
**1w**	6-Methoxy	2'-Methoxy	10.71	17.04	3.39	NI	5.58	13.05
**1x**	6-Methoxy	3'-Methoxy	8.72	9.79	NI	NI	0.09	0.98
**Sulfuretin**	6-Hydoxy	3',4'-Dihydoxy	6.04	3.30	NI	NI	8.36	6.56

Table S8. % Growth inhibition of diverse prostate cancer cell lines triggered by 10 μM concentrations of sulfuretin and its prepared analogs (**1a**–**x**).

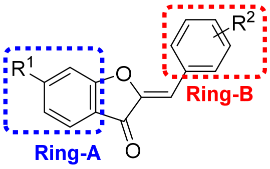

1a–x

Comp	R¹	R²	PC3	DU145
**1a**	6-Hydoxy	2'-Hydoxy	NI	NI
**1b**	6-Hydoxy	3'-Hydoxy	NI	NI
**1c**	6-Hydoxy	4'-Hydoxy	NI	NI
**1d**	6-Hydoxy	2',4'-Dihydoxy	1.60	NI
**1e**	6-Hydoxy	2',5'-Dihydoxy	NI	NI
**1f**	6-Hydoxy	3',5'-Dihydoxy	0.38	NI
**1g**	6-Hydoxy	3',4',5'-Trihydoxy	3.49	NI
**1h**	6-Hydoxy	2'-Methoxy	NI	NI
**1i**	6-Hydoxy	3'-Methoxy	NI	6.41
**1j**	6-Hydoxy	4'-Methoxy	NI	NI
**1k**	6-Hydoxy	2',3'-Dimethoxy	NI	NI
**1l**	6-Hydoxy	2',5'-Dimethoxy	4.50	22.55
**1m**	6-Hydoxy	3',4'-Dimethoxy	0.51	NI
**1n**	6-Hydoxy	3',5'-Dimethoxy	9.19	37.22
**1o**	6-Hydoxy	2',3',4'-Trimethoxy	NI	NI
**1p**	6-Hydoxy	4'-Methoxymethoxy	1.55	NI
**1q**	6-Methoxy	2'-Hydoxy	NI	NI
**1r**	6-Methoxy	3'-Hydoxy	NI	NI
**1s**	6-Methoxy	4'-Hydoxy	NI	NI
**1t**	6-Methoxy	2',3'-Dihydoxy	10.05	32.09
**1u**	6-Methoxy	2',4'-Dihydoxy	NI	NI
**1v**	6-Methoxy	3',4'-Dihydoxy	5.56	7.73
**1w**	6-Methoxy	2'-Methoxy	0.44	1.50
**1x**	6-Methoxy	3'-Methoxy	NI	NI
**Sulfuretin**	6-Hydoxy	3',4'-Dihydoxy	NI	NI

Table S9. % Growth inhibition of diverse breast cancer cell lines triggered by 10 µM concentrations of sulfuretin and its prepared analogs (**1a**–**x**).

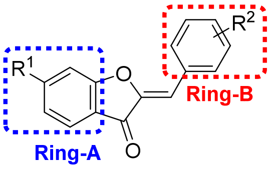

1a–x

Comp	R¹	R²	MCF7	BT549	MDAMB231	MDAMB468
**1a**	6-Hydoxy	2'-Hydoxy	NI	NI	NI	NI
**1b**	6-Hydoxy	3'-Hydoxy	NI	NI	NI	NI
**1c**	6-Hydoxy	4'-Hydoxy	NI	NI	NI	1.05
**1d**	6-Hydoxy	2',4'-Dihydoxy	NI	NI	NI	1.93
**1e**	6-Hydoxy	2',5'-Dihydoxy	NI	NI	NI	NI
**1f**	6-Hydoxy	3',5'-Dihydoxy	NI	NI	NI	NI
**1g**	6-Hydoxy	3',4',5'-Trihydoxy	2.60	5.91	NI	NI
**1h**	6-Hydoxy	2'-Methoxy	0.99	NI	NI	NI
**1i**	6-Hydoxy	3'-Methoxy	NI	20.43	NI	NI
**1j**	6-Hydoxy	4'-Methoxy	NI	7.25	NI	NI
**1k**	6-Hydoxy	2',3'-Dimethoxy	NI	NI	6.87	NI
**1l**	6-Hydoxy	2',5'-Dimethoxy	5.77	20.23	NI	NI
**1m**	6-Hydoxy	3',4'-Dimethoxy	NI	NI	NI	NI
**1n**	6-Hydoxy	3',5'-Dimethoxy	22.80	45.64	7.24	13.57
**1o**	6-Hydoxy	2',3',4'-Trimethoxy	NI	NI	NI	NI
**1p**	6-Hydoxy	4'-Methoxymethoxy	NI	NI	1.70	NI
**1q**	6-Methoxy	2'-Hydoxy	17.10	9.74	2.51	NI
**1r**	6-Methoxy	3'-Hydoxy	26.79	4.51	3.89	0.43
**1s**	6-Methoxy	4'-Hydoxy	NI	21.09	NI	NI
**1t**	6-Methoxy	2',3'-Dihydoxy	48.96	37.85	37.51	19.42
**1u**	6-Methoxy	2',4'-Dihydoxy	NI	7.28	NI	3.77
**1v**	6-Methoxy	3',4'-Dihydoxy	NI	16.70	16.52	5.96
**1w**	6-Methoxy	2'-Methoxy	6.52	9.03	NI	24.09
**1x**	6-Methoxy	3'-Methoxy	NI	NI	NI	1.90
**Sulfuretin**	6-Hydoxy	3',4'-Dihydoxy	NI	NI	3.89	1.16
